# Semaphorin 3C (Sema3C) reshapes stromal microenvironment to promote hepatocellular carcinoma progression

**DOI:** 10.1038/s41392-024-01887-0

**Published:** 2024-07-03

**Authors:** Hao Peng, Meng Yang, Kun Feng, Qingpeng Lv, Yewei Zhang

**Affiliations:** 1 Medical School, Southeast University, Nanjing, 210009 China; 2 Department of Ultrasound, State Key Laboratory of Complex Severe and Rare Diseases, Peking Union Medical College Hospital, Chinese Academy of Medical. Sciences, Peking Union Medical College, Beijing, 100730 China; 3 Hepatopancreatobiliary Center, The Second Affiliated Hospital of Nanjing Medical University, Nanjing, 210009 China

**Keywords:** Cancer microenvironment, Cancer stem cells

## Abstract

More than 90% of hepatocellular carcinoma (HCC) cases develop in the presence of fibrosis or cirrhosis, making the tumor microenvironment (TME) of HCC distinctive due to the intricate interplay between cancer-associated fibroblasts (CAFs) and cancer stem cells (CSCs), which collectively regulate HCC progression. However, the mechanisms through which CSCs orchestrate the dynamics of the tumor stroma during HCC development remain elusive. Our study unveils a significant upregulation of Sema3C in fibrotic liver, HCC tissues, peripheral blood of HCC patients, as well as sorafenib-resistant tissues and cells, with its overexpression correlating with the acquisition of stemness properties in HCC. We further identify NRP1 and ITGB1 as pivotal functional receptors of Sema3C, activating downstream AKT/Gli1/c-Myc signaling pathways to bolster HCC self-renewal and tumor initiation. Additionally, HCC cells-derived Sema3C facilitated extracellular matrix (ECM) contraction and collagen deposition in vivo, while also promoting the proliferation and activation of hepatic stellate cells (HSCs). Mechanistically, Sema3C interacted with NRP1 and ITGB1 in HSCs, activating downstream NF-kB signaling, thereby stimulating the release of IL-6 and upregulating HMGCR expression, consequently enhancing cholesterol synthesis in HSCs. Furthermore, CAF-secreted TGF-β1 activates AP1 signaling to augment Sema3C expression in HCC cells, establishing a positive feedback loop that accelerates HCC progression. Notably, blockade of Sema3C effectively inhibits tumor growth and sensitizes HCC cells to sorafenib in vivo. In sum, our findings spotlight Sema3C as a novel biomarker facilitating the crosstalk between CSCs and stroma during hepatocarcinogenesis, thereby offering a promising avenue for enhancing treatment efficacy and overcoming drug resistance in HCC.

## Introduction

Hepatocellular carcinoma (HCC), the most common malignant tumor with a poor prognosis, has a unique premalignant environment (PME) characterized by the fact that over 90% of cases arise from fibrotic or cirrhotic livers.^1,2^ Hepatic stellate cells (HSCs) are situated in the subendothelial space of Disse, nestled between hepatic sinusoidal endothelial cells and hepatocytes, and play a crucial role in liver physiology and fibrosis. During chronic liver injury, quiescent HSCs undergo trans-differentiation into activated, proliferative myofibroblasts, which are the primary source of extracellular matrix (ECM) components.^3^ HSCs are a part of the cell components in the tumor microenvironment. On the one hand, HSCs promote hepatocarcinogenesis by strongly interacting with hepatocytes primarily in the PME.^4,5^ On the other hand, it can also interact with non-cellular components to affect tumor progression, such as the secretion and up-regulation of matrix metalloproteinases (MMP) and other proteins to promote fiber formation and ECM remodeling.^6^ A recent study has shown that there exist two distinct HSC subpopulations in the fibrotic liver, myofibroblastic HSCs (myHSCs) promote hepatocarcinogenesis, while the cytokine- and growth-factor-expressing HSCs (cyHSCs) suppress HCC. The imbalance between myHSCs and cyHSCs contributes to an increased risk for HCC development, and this imbalance is spatially localized to a specific region.^5^ In the HCC region, as a major cellular component of HCC stromal microenvironment, cancer-associated fibroblasts (CAFs) are believed to originate from HSCs.^7^ CAFs support tumorigenesis by directly stimulating tumor cell proliferation, promoting angiogenesis and reshaping the microenvironment. These functions are performed through ligand-receptor interactions, the release of growth factors and inflammatory cytokines and/or the promotion of deposition of ECM components.^8^ Additionally, the spatial distribution of CAFs within the tumor may significantly influence tumor progression. Studies have shown that CAFs in direct contact with tumor cells activate TGFβ signaling and collagen deposition, while CAFs farther away from the tumor promote hyaluronic acid deposition and reshape the TME by secreting IL-6 and other inflammatory mediators.^9^ Moreover, emerging evidence suggests that CAFs promote chemoresistance primarily by disrupting drug delivery and biochemical signaling within tumor cells. HCC organoids co-cultured with CAFs or its conditioned medium can make tumor cells resistant to anti-tumor drugs such as sorafenib, regafenib, and 5-fluorouracil.^9^ It is worth noting that PME-localized HSCs and TME-localized CAFs affect HCC development through distinct mechanisms, with the former mainly influencing the early-stage lesions in the persistently damaged liver, while the latter mainly accelerating the malignant progression of advanced HCC.^5^ However, the mechanism by which the TME exacerbates the imbalance between myHSCs and cyHSCs and promotes the conversion of HSCs to CAFs is not yet clear.

Cancer stem cells (CSCs), as a distinctive subgroup of tumor cells, have the capacity to self-renew and differentiate into other cell types. These characteristics enable CSCs to resist chemotherapy, thereby facilitating tumor recurrence.^10^ Many cell surface proteins have been identified as CSCs markers of HCC, such as CD44, CD133, EpCAM, CD34, and CD24, as well as transcription factors/intracellular markers, such as SOX2, OCT4, NANOG. Most of the biomarkers expressed in CSCs are not specific and can also be expressed in normal embryonic stem cells or normal tissues.^11^ In addition, CSCs are regulated by multiple signaling pathways to maintain their self-renewal capacity. Key pathways include Wnt/β-catenin, Hedgehog, and PI3K/AKT, which are crucial for maintaining stemness and promoting tumorigenesis. Activation of these and other pathways is believed to lead to the initial formation of CSCs.^12^ The self-renewal maintenance of CSCs also depends on the support of the stromal microenvironment, and mounting evidence has shown that CAFs maintain the properties of CSCs by secreting proteins or extracellular vesicles.^10^ Furthermore, CSCs can also recruit or stimulate stromal cells within niches to reshape a more supportive TME.^13^ However, at present, there are few studies on the regulation of CAFs by CSCs in HCC.

To elucidate the roles of the interaction between tumor stroma and CSCs in HCC tumorigenesis, we identified Semaphorin 3C (Sema3C), as a highly upregulated protein in fibrosis and HCC tissues. More than 20 Semaphorin proteins have been discovered, among which only class 3 are secreted signaling proteins in vertebrates. Sema3C functions as an axon attractant for migrating cortical axons and promotes axon growth in human dopaminergic neurons, guiding motor neurons to specific targets.^14^ A previous study suggested that upregulated Sema3C is significantly correlated with tumor size and poor prognosis in HCC.^15^ However, the underlying mechanism of Sema3C up-regulation in HCC and whether it is implicated in the remodeling of TME remain unclear.

In our research, we discovered that Sema3C promotes the stemness maintenance of HCC in an autocrine way. In addition, Sema3C reshapes ECM and promotes HSCs activation and malignant transformation towards CAFs via paracrine way. In turn, TME-localized CAFs stimulate the Sema3C expression through the secretion of TGF-β1. Therefore, Sema3C may serve as a key biomarker to mediate the communication between the CSCs and tumor stroma, forming a vicious cycle in the development of HCC.

## Results

### Highly expressed Sema3C is correlated with stemness features in HCC

**Fig. 1:**
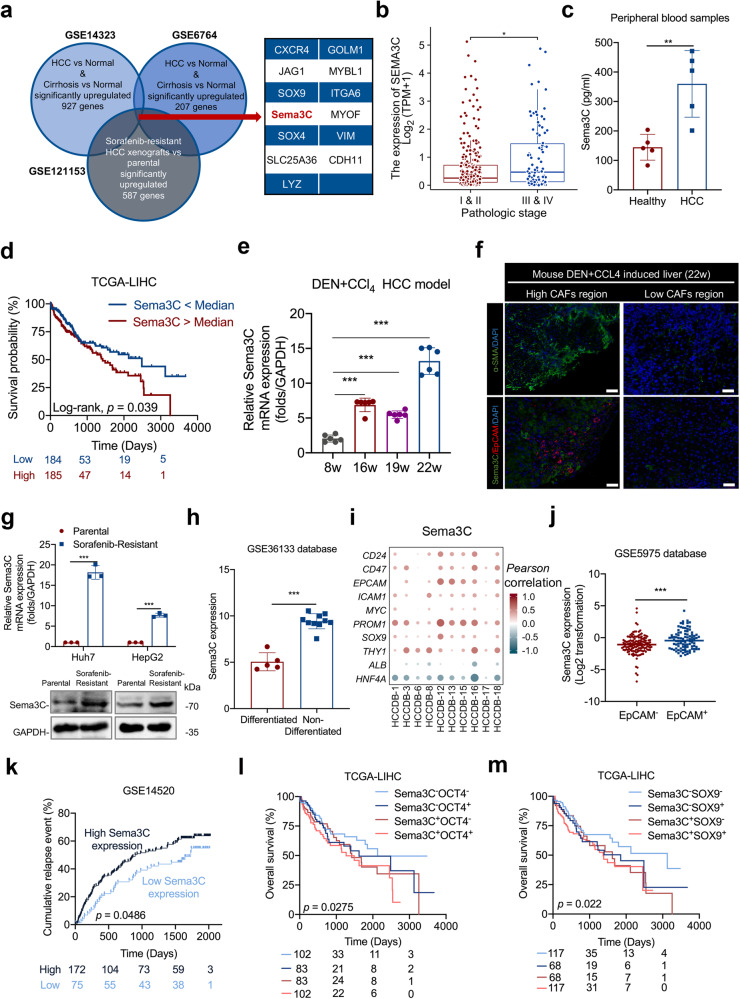
Highly expressed Sema3C is correlated with stemness features in HCC. **a** Identification of Sema3C with integrative analysis of public datasets, including genes up-regulated in both cirrhosis and HCC (GSE14323 and GSE6764) and in sorafenib-resistant HCC xenografts (GSE121153). **b** Correlation analysis between Sema3C expression and pathological stage. **c** The expression of Sema3C in peripheral blood of HCC patients and healthy individuals was determined by ELISA, n = 5. **d** Kaplan‒Meier survival analysis for overall survival in TCGA-LIHC dataset based on Sema3C median expression. **e** qRT-RCR analysis of Sema3C expression in DEN+CCl_4_-induced HCC model at various stages. 8w indicates normal liver, 16-19w indicates fibrotic liver, and 22w indicates HCC. **f** IF staining for Sema3C and EpCAM in DEN+CCl_4_-induced HCC tissues (22w). Cell nuclei were counterstained with DAPI. Scale bar, 50 μm. **g** The mRNA and protein expression of Sema3C in sorafenib-sensitive and sorafenib-resistant Huh7 and HepG2 cells. **h** Sema3C is highly upregulated in non-differentiated HCC cell lines compared to differentiated HCC cell lines from the GSE36133 database. **i** Correlation between Sema3C expression and stemness-associated genes in HCC using the HCCDB datasets. The dots in the figure indicate p < 0.05. The color intensity and size of the circle are proportional to the correlation coefficients. **j** Expression of Sema3C in EpCAM^-^ and EpCAM^+^ cells from the GSE5975 database. **k** Cumulative relapse event analysis to predict HCC recurrence based on Sema3C median expression from the GSE14520 dataset. Kaplan‒Meier survival analysis for overall survival in TCGA-LIHC dataset based on Sema3C expression and OCT4^+^ (**l**) or SOX9^+^ (**m**) expression, respectively. Data are presented as means ± SD. *p < 0.05, **p < 0.01, ***p < 0.001

To explore the mechanism of interaction between CSCs and tumor stroma, we screened out genes that were highly expressed in cirrhosis and HCC tissues compared to normal liver tissues in GSE14323 and GSE6764 databases, and intersected with genes that were elevated in sorafenib-resistant HCC xenografts based on GSE121153 dataset to obtain 13 common genes (Fig. 1a). Among the 13 genes, we looked for secretory proteins that may mediate intercellular communication. Previous studies have reported that Sema3C, as a secreted glycoprotein, was elevated in HCC tissues and correlated with tumor size, portal vein embolization, and metastasis.^15,16^ Consistently, we found that Sema3C expression was higher in patients with stage III&IV than in patients with stage I&II (Fig. 1b). To detect the differential expression of Sema3C in circulatory levels between HCC patients and healthy individuals, we collected peripheral blood of 5 HCC patients and healthy people respectively and found that the concentration of Sema3C in HCC patients was higher than that of healthy individuals (Fig. 1c). By analyzing the data of TCGA-LIHC, HCC patients with elevated Sema3C expression have a worse prognosis compared to those with low Sema3C expression (Fig. 1d). Furthermore, we demonstrated that Sema3C expression was greatly enhanced at the fibrotic and advanced stages of DEN+CCl_4_-induced HCC models (Fig. 1e). Meanwhile, based on this HCC model, we also preliminarily evaluated the association of Sema3C with stemness. Tissue immunofluorescence (IF) showed that Sema3C and EpCAM expression were highly expressed in areas with high infiltration of CAFs, while low expression in areas with low abundance of CAFs (Fig. 1f). Previously, we constructed two sorafenib-resistant cell lines (Huh7 and HepG2),^17^ the results showed that Sema3C expression was significantly increased in sorafenib-resistant HCC cells (Fig. 1g).

To further investigate the relationship between Sema3C and stemness, we analyzed its expression in both differentiated and non-differentiated HCC cell lines using the GSE36133 dataset, revealing higher expression in the latter. (Fig. 1h). In addition, by analyzing the correlations between the Sema3C and multiple stemness-related genes using the HCCDB databases, we discovered Sema3C was positively associated with CSCs markers, such as *CD24, CD47, EPCAM, ICAM1, c-Myc, PROM1* (encoding CD133), *SOX9*, and *THY1* (encoding CD90), while negatively correlated with hepatic lineage genes, such as *ALB* and *HNF4A* (Fig. 1i). Then, we compared Sema3C expression in EpCAM^+^ cells and EpCAM^-^ cells in the GSE5975 database and found that Sema3C was up-regulated in EpCAM^+^ cells (liver CSCs) (Fig. 1j). Given that CSCs contribute to tumor recurrence, the impact of Sema3C on HCC relapse was examined using the GSE14520 dataset. The results showed that patients with highly expressed Sema3C had more HCC recurrence events than patients with low Sema3C expression (Fig. 1k). Besides, the survival analysis, stratified by the expression of Sema3C and stemness-related genes (OCT4 and SOX9), revealed that HCC patients expressing both Sema3C and OCT4/SOX9 had the worst prognosis (Fig. 1l, m). Altogether, these results suggested that Sema3C was up-regulated during HCC progression and was associated with HCC stemness.

### Sema3C promotes stemness maintenance and initiation in HCC

**Fig. 2:**
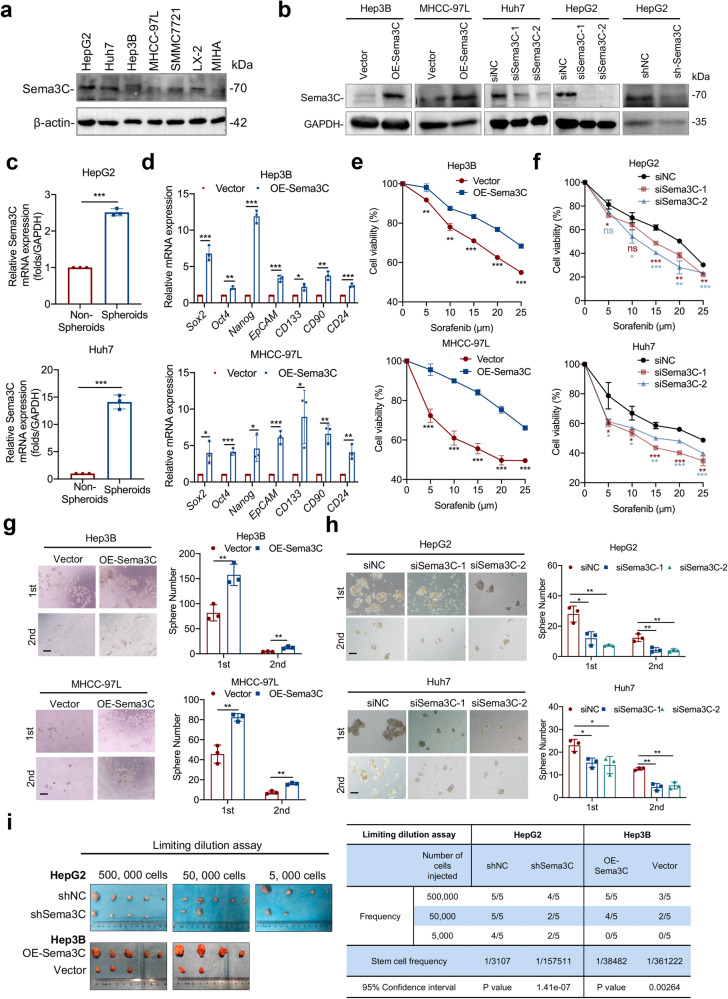
Sema3C promotes HCC stemness and initiation. **a** Sema3C protein expression levels in various HCC cell lines, hepatic stellate cells (LX-2) and normal liver cells (MIHA) were detected by Western blotting. **b** Sema3C protein expression levels were determined by Western blotting in Sema3C overexpressed and knockdown HCC cells. **c** Sema3C mRNA expression level in spheroids and non-spheroids cells of HepG2 and Huh7 cells. **d** The mRNA expression of stemness-associated genes in Sema3C-overexpressing HCC and control cells was determined by qRT-PCR analysis. The effect of Sema3C overexpression (**e**) or knockdown (**f**) on HCC cell chemoresistance was assessed by an MTT assay. The effect of Sema3C overexpression (**g**) or knockdown (**h**) on HCC cells self-renewal was evaluated by sphere formation assay. Scale bar, 100 μm. **i** Limiting dilution assay was conducted using HepG2 cells with Sema3C knockdown (shSema3C) or Hep3B cells infected with lentivirus-OE-Sema3C or empty vector in BALB/c nude mice (n = 5 per group). Student’s *t* test was used for comparing two groups and extreme limiting dilution analysis (ELDA) was used for limiting dilution assay. OE overexpression, NC nontarget control. Data are presented as means ± SD. *p < 0.05, **p < 0.01, ***p < 0.001

To further explore the effect of Sema3C on the stemness phenotype of HCC, we compared the protein levels of Sema3C across multiple HCC cell lines. Our findings revealed elevated Sema3C expression in HCC cell lines compared to normal liver cell lines, and Sema3C was also expressed in hepatic stellate cells (LX-2) to a certain extent (Fig. 2a). In addition, we analyzed Sema3C expression variations across cell types in HCC using published single-cell datasets (GSE146115, GSE146409, and GSE166635). The results indicated that Sema3C was predominantly expressed in tumor cells and, to a lesser extent, in stromal cells, while it was rarely expressed in normal hepatocytes or endothelial cells, consistent with our in vitro cell line results (Supplementary Fig. 1a). Then, Hep3B and MHCC-97L were selected for Sema3C overexpression, while HepG2 and Huh7 were chosen for Sema3C knockdown (Fig. 2b). To determine Sema3C expression in CSC populations, we compared the protein levels of Sema3C between spheroids and non-spheroids. The results showed that Sema3C expression was significantly higher in spheroids than in non-spheroids (Fig. 2c). In line with bioinformatics analysis, Sema3C overexpression up-regulated the expression of stemness-related genes (*Sox2, Oct4, Nanog, EpCAM, CD133, CD90, and CD24*) in HCC cells when compared to empty vector controls (Fig. 2d). To further investigate whether Sema3C functionally contributes to drug resistance, self-renewal, and tumorigenesis, we conducted MTT and sphere formation assays. Sema3C overexpression in HCC cells significantly increased resistance to sorafenib, while Sema3C knockdown led to a marked decrease in resistance. (Fig. 2e, f). Meanwhile, HCC cells overexpressing Sema3C showed increased spheroid formation, while Sema3C knockdown cells had reduced spheroid formation (Fig. 2g, h). Additionally, colony formation experiments revealed that Sema3C overexpression promoted HCC proliferation, whereas Sema3C knockdown inhibited cell proliferation (Supplementary Fig. 1b, c). We also found that apoptosis was significantly inhibited in Sema3C-overexpressing HCC cells and enhanced in Sema3C-knockdown HCC cells (Supplementary Fig. 1d, e). Transwell assays further demonstrated that Sema3C overexpression promoted cell invasion and migration, whereas Sema3C knockdown attenuated cell invasion and migration (Supplementary Fig. 1f, g).

To evaluate the impact of Sema3C on tumor-initiating ability in vivo, a limiting dilution assay was conducted by subcutaneously injecting 5 × 10^5^, 5 × 10^4^, and 5 × 10^3^ HCC cells into nude mice. Hep3B cells overexpressing Sema3C showed a significantly higher tumor-initiating capacity compared to control cells. In contrast, Sema3C knockdown HCC cells resulted in a significantly reduced number of tumors compared to controls (Fig. 2i). Next, to verify the stemness maintenance role of secreted Sema3C in the TME, we conducted a series of rescue experiments. On the basis of endogenous Sema3C knockdown, recombinant human Sema3C (rhSema3C) was used to stimulate HCC cells. Transwell assay found that addition of rhSema3C could reverse the impact of Sema3C knockdown on invasion and migration of HCC cells (Supplementary Fig. 2a). MTT assay showed that the rhSema3C could reverse the sensitivity to sorafenib in HCC cells with Sema3C knockdown (Supplementary Fig. 2b). In addition, in vitro sphere formation also confirmed that secreted Sema3C could restore the spheroid formation of HCC cells upon Sema3C knockdown (Supplementary Fig. 2c). In summary, these findings demonstrate that Sema3C plays a critical role in enhancing HCC stemness, chemoresistance, and tumor initiation.

### Sema3C maintains HCC stemness via a dysregulated AKT/Gli1/c-Myc signaling axis

**Fig. 3:**
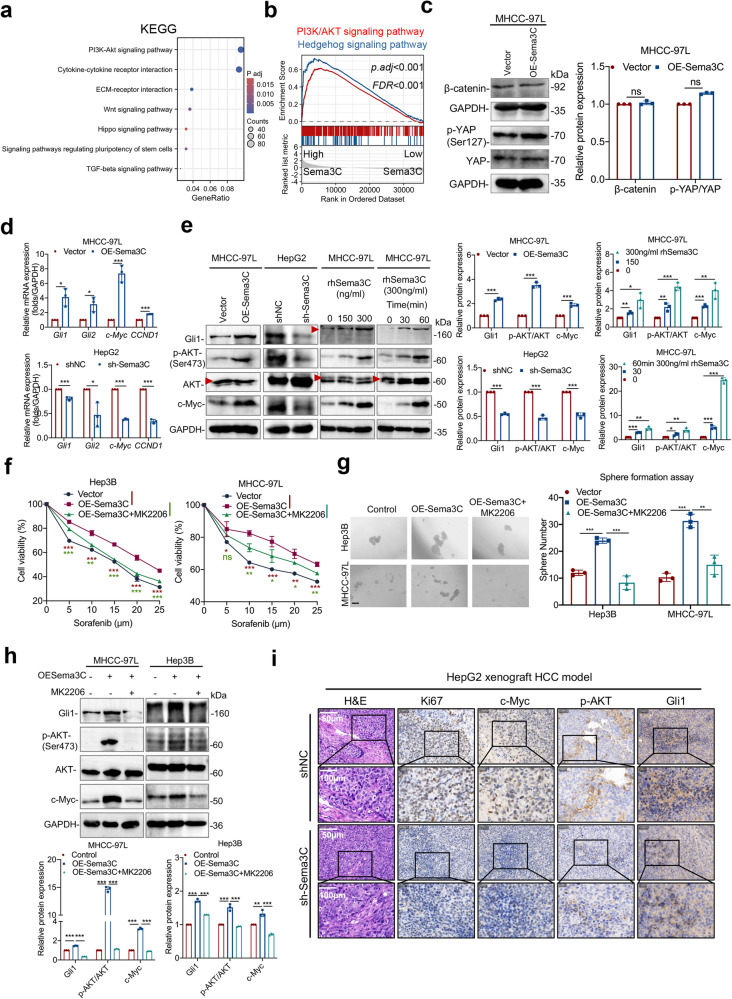
Sema3C drives HCC initiation via a dysregulated AKT/Gli1/c-Myc signaling axis. **a** KEGG pathway analysis found Sema3C to be highly correlated with PI3K-AKT signaling in HCC patients based on the TCGA-LIHC database. **b** GSEA identified an enrichment of genes involved in PI3K/AKT and Hedgehog signaling pathways in the high-Sema3C-expressing HCC group. **c** Western blotting analyses of the β-catenin and phosphorylated-YAP levels in Sema3C-overexpressing MHCC-97L cells. **d** The mRNA expression of *Gli1, Gli2, c-Myc*, and *CCND1* in MHCC-97L cells transfected with OE-Sema3C or vector control and HepG2 cells transfected with sh-Sema3C or NC. **e** The protein expression levels of total AKT, p-AKT, Gli1, and c-Myc in MHCC-97L cells and HepG2 cells. Sema3C-overexpressing Hep3B and MHCC-97L cells were treated with MK2206 (AKT inhibitor) as indicated, and the cell viability was analyzed by an MTT assay (**f**), the ability of self-renewal was determined by a sphere formation assay, scale bar, 100 μm (**g**); the levels of Gli1, p-AKT, AKT, and c-Myc were determined by western blotting (**h**). **i** H&E and IHC staining for Ki67, c-Myc, p-AKT (Ser473) and Gli1 in HepG2 xenograft tumors. NC nontarget control; H&E hematoxylin-eosin; IHC immunohistochemistry; Data are presented as means ± SD. ns, not significantly; *p < 0.05, **p < 0.01, ***p < 0.001

To investigate the downstream mechanisms by which Sema3C maintains HCC stemness, pathway enrichment analysis based on the TCGA dataset was conducted. The result revealed a strong association between Sema3C and signaling pathways regulating stem cell pluripotency, consistent with our above findings. Additionally, significant KEGG enrichment was observed in the PI3K-AKT, Wnt, and Hippo signaling pathways (Fig. 3a). GSEA analysis also revealed that the PI3K-AKT and Hedgehog signaling pathways were highly enriched in HCC samples with elevated Sema3C expression (Fig. 3b). Given the role of PI3K/AKT, Wnt, Hippo, and Hedgehog pathways in stemness regulation, we detected key molecules within these pathways. Results suggested that Sema3C overexpression did not alter β-catenin or phosphorylated-YAP (p-YAP) protein levels (Fig. 3c). However, the mRNA levels of *Gli1, Gli2, c-Myc*, and *CCND1* were increased in overexpressed-Sema3C HCC cells, and on the contrary, the levels of these target genes were decreased upon Sema3C knockdown (Fig. 3d). Consistently, western blot analysis confirmed that key mediators of the AKT and Hedgehog pathways—p-AKT, Gli1, and c-Myc—were highly expressed in Sema3C-overexpressing MHCC-97L HCC cells. Conversely, knockdown Sema3C in HepG2 cells attenuated the activated signaling. Furthermore, stimulation of MHCC-97L cells with varying doses of rhSema3C or at different time points revealed a dose- and time-dependent activation of the Gli1/AKT pathway (Fig. 3e). To further validate the significance of AKT/Gli1 signaling in Sema3C-driven HCC stemness, functional rescue experiments were performed in which a specific AKT inhibitor MK2206 was introduced into the Sema3C overexpressing HCC cells. MK2206 attenuated the stemness features induced by Sema3C overexpression, as evidenced by reduced chemoresistance in HCC cells (Fig. 3f), sphere-formation (Fig. 3g), migration, and invasion (Supplementary Fig. 2d). Previous study has demonstrated that the PI3K/AKT pathway could modulate the Hedgehog signaling pathway in renal cell carcinoma.^18^ Our findings indicated that MK2206 treatment blocked Sema3C-induced Gli1 protein expression in HCC cells, suggesting Sema3C could act as a mediator in potentiating AKT signaling activation of the Hedgehog pathway in HCC (Fig. 3h). This was further validated in an in vivo HCC model, where HepG2 cells with or without Sema3C knockdown were subcutaneously injected into nude mice. Upon Sema3C knockdown, the immunohistochemistry (IHC) staining revealed a decreased Ki67 expression as well as down-regulation of activated p-AKT, c-Myc, and Gli1 versus control tumors (Fig. 3I). Collectively, Sema3C-mediated activation of AKT and Gli1 signaling pathways promoted stemness maintenance of HCC.

### Sema3C maintain HCC stemness via NRP1 and ITGB1

**Fig. 4:**
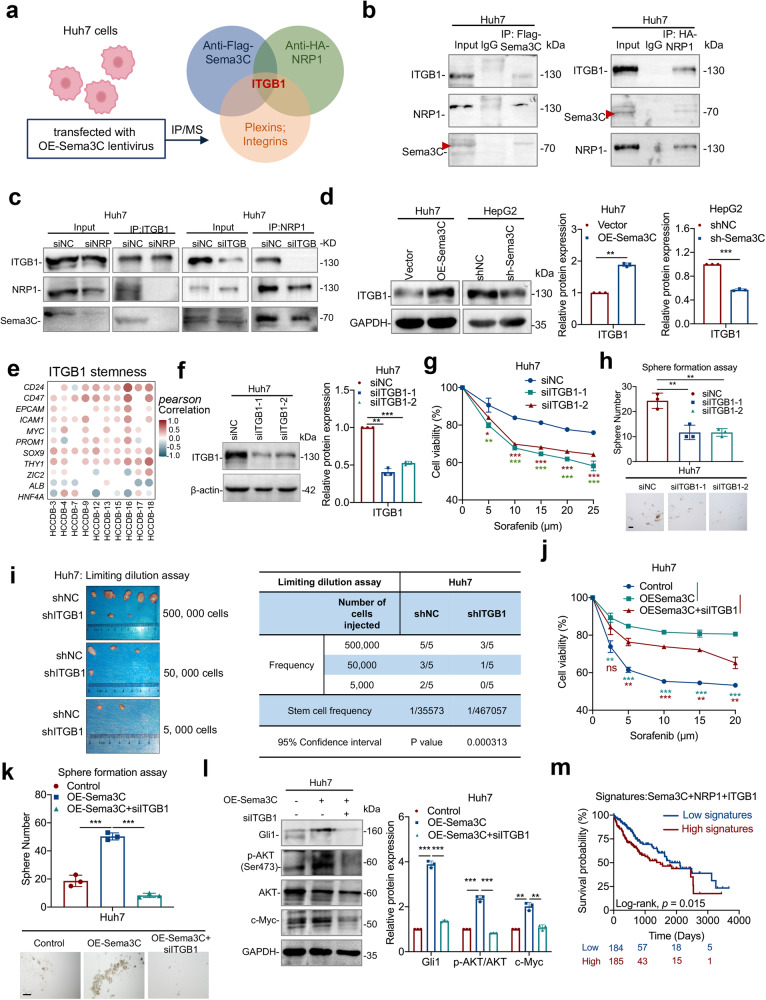
Sema3C drives stemness maintenance and signaling via NRP1 and ITGB1. **a** Scheme displaying the procedure used for identifying the potential receptors that can bind to Sema3C and NRP1. **b** Co-IP analysis for validation of ITGB1 as a binding protein of Sema3C or NRP1 in Huh7 cells. **c** NRP1 and ITGB1 were knocked down in Huh7 cells respectively, and the interaction between Sema3C, NRP1 and ITGB1 was detected by Co-IP. **d** ITGB1 expression was detected in HCC cells when Sema3C was overexpressed or knocked down. **e** Correlation between ITGB1 expression and stemness-associated genes in HCC using the HCCDB datasets. The dots in the figure indicate p < 0.05. The color intensity and size of the circle are proportional to the correlation coefficients. **f** The expression levels of ITGB1 were determined by western blotting in ITGB1 knockdown Huh7 cells. Huh7 cells transfected with siITGB1. The chemoresistant effect was investigated by an MTT assay (**g**). The ability of self-renewal was determined by a sphere formation assay, scale bar, 100 μm (**h**). **i** Limiting dilution assay of Huh7 cells with or without ITGB1 knockdown (shITGB1) in BALB/c nude mice (n = 5 per group). Huh7 cells were transfected with OE-Sema3C or siITGB1. The chemoresistant effect was investigated by an MTT assay (**j**). The ability of self-renewal was determined by a sphere formation assay, scale bar, 100 μm (**k**). The protein expression levels of Gli1, p-AKT, AKT, and c-Myc were validated by western blotting (**l**). **m** Kaplan–Meier survival analysis for overall survival of HCC patients segregated according to high or low gene signatures for Sema3C + NRP1 + ITGB1. Co-IP Coimmunoprecipitation. OE overexpression. NC nontarget control. Data are presented as means ± SD. ns, not significantly *p < 0.05, **p < 0.01, ***p < 0.001

Previous studies have shown that Sema3C could bind to the neuropilins (Nrp1 or Nrp2), to mediate downstream signal transduction.^19^ Moreover, NRP1 was up-regulated in HCC and promoted the expansion of CSCs and tumor growth.^20,21^ To this end, we examined whether NRP1 mediated the stemness maintenance for Sema3C in HCC. MHCC-97L and Hep3B cells were treated with NRP1-targeting siRNA (Supplementary Fig. 3a, b), and results showed that knockdown of NRP1 attenuated Sema3C-induced chemoresistance, self-renewal, migration, and invasion of HCC cells compared to control cells (Supplementary Fig. 3c–e). Furthermore, we examined whether Sema3C-activated AKT and Gli1 signaling depended on NRP1 binding. Our results revealed that Sema3C-induced phosphorylation of AKT, and an increase of Gli1 and c-Myc expression were reversed by NRP1 knockdown (Supplementary Fig. 3f). NRP1 binds to a variety of downstream receptors, including the plexins and the integrins family.^16^ To identify co-receptors mediating stemness maintenance after Sema3C binds to NRP1, Sema3C and NRP1 were overexpressed in Huh7 cells, co-immunoprecipitation combined with mass spectrometry (IP/MS) revealed that only ITGB1, which belonged to the integrin family, could bind to both Sema3C and NRP1 (Fig. 4a, b, Supplementary Fig. 4a). Analysis of the TCGA-LIHC database showed a positive correlation between Sema3C and ITGB1 expression in HCC (Supplementary Fig. 4b). Co-immunoprecipitation (Co-IP) assays in Huh7 cells also confirmed the binding of Sema3C, NRP1, and ITGB1 (Fig. 4b). Simulated molecular docking results showed that VWFA domain (amino acids 140-378) of ITGB1 bound to Sema3C (Supplementary Fig. 4c). Moreover, NRP1 knockdown in Huh7 cells inhibited ITGB1 from binding to Sema3C, while NRP1 still bound Sema3C after ITGB1 knockdown, indicating that NRP1 acted as a ligand-binding receptor, facilitating the formation of a complex with Sema3C and ITGB1 in HCC cells (Fig. 4c). Furthermore, ITGB1 expression was significantly elevated in Sema3C-overexpressing Huh7 cells but decreased in Sema3C-knockdown HepG2 cells (Fig. 4d).

Previous studies have shown that ITGB1 facilitates sorafenib resistance and tumor formation of HCC.^22,23^ Our study investigated the role of ITGB1 in HCC stemness regulation and found a positive correlation between ITGB1 expression and multiple stemness-related genes in the HCCDB database (Fig. 4e). siRNA was used to knock down ITGB1 in Huh7 cells (Fig. 4f), and results revealed that ITGB1 knockdown significantly inhibited chemoresistance, sphere-forming ability, migration, and invasion (Fig. 4g, h, Supplementary Fig. 4d). Concordantly, a subcutaneous tumor model of HCC showed that ITGB1 knockdown led to a significant reduction in tumor incidence rate (Fig. 4i). To characterize the role of ITGB1 in Sema3C-directed stemness, we transfected Huh7 cells with OE-Sema3C, with or without si-ITGB1. ITGB1 knockdown abrogated Sema3C-induced chemoresistance, self-renewal, migration, and invasion (Fig. 4j, k, Supplementary Fig. 4e). Moreover, silencing ITGB1 also antagonized Sema3C-induced upregulation of AKT phosphorylation, GlI1, and c-Myc expression (Fig. 4l). Additionally, NRP1 or ITGB1 knockdown in HCC cells weakened the stemness effect of rhSema3C, suggesting that rhSema3C relied on NRP1 and ITGB1 to exert its function (Supplementary Fig. 4f-h). Analysis of TCGA-LIHC data showed that HCC patients with high Sema3C, NRP1, and ITGB1 expression signatures had a worse prognosis (Fig. 4m). These results suggest that Sema3C regulates HCC stemness through the NRP1/ITGB1 axis.

### HCC cells-derived Sema3C promotes ECM remodeling and HSCs activation

**Fig. 5:**
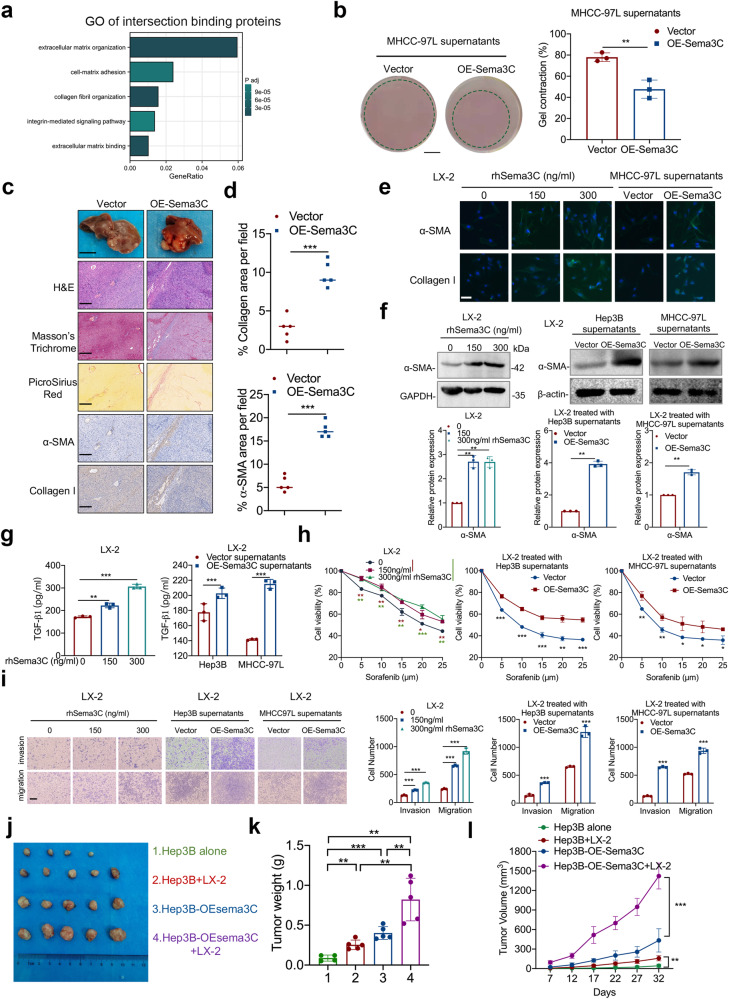
Sema3C is involved in the ECM remodeling and induces the HSCs activation. **a** GO analysis based on intersection proteins binding to Sema3C and NRP1. **b** Representative images of collagen gel contraction assays with MHCC-97L cells in either MHCC-97L supernatants or OE-Sema3C-MHCC-97L supernatants. Graph quantifying the change in the percentages of gel contraction by MHCC-97L cells in different conditions. Scale bar, 5 mm. **c** Representative images showing H&E, Masson’s trichrome and picrosirius red staining, and IHC staining for α-SMA and collagen I expression in serial sections of orthotopic liver xenograft tumors of BALB/C nude mice injected with MHCC-97L-Vector or MHCC-97L-OE-Sema3C cells. Gross tissue image scale bar, 1 cm, microscope image scale bar, 200 μm. **d** Percent of collagen fibers and α-SMA per field were counted in at least three random fields per animal, (n = 5). **e** Representative immunofluorescence images for α-SMA and collagen I in HSCs treated with dosage rhSema3C or supernatants of different Sema3C expression levels. Scale bar, 50 μm. LX-2 cells were treated with a gradient dose of rhSema3C or supernatants from Sema3C-overexpressed Hep3B and MHCC-97L cells. The α-SMA expression levels were detected by western blotting analysis (**f**). The TGF-β1 secretion was examined by ELISA assay (**g**). The chemoresistance ability was reflected by an MTT assay (**h**). The ability of migration and invasion was performed by Transwell assays, scale bar, 200 μm (**i**). 5 × 10^5^ Hep3B cells or OE-Sema3C Hep3B cells were injected subcutaneously into nude mice alone or mixed with LX-2 cells in a 1:1 ratio. The mice were sacrificed, and the xenograft tumors were excised 32 days after inoculation (**j**). The bar chart showed the tumor weight in each treatment group (**k**). The tumor volumes were monitored for 32 days (**l**). n = 5 per group. Data are presented as means ± SD. *p < 0.05, **p < 0.01, ***p < 0.001

As axon guidance molecules, semaphorins establish and maintain effective communication links between axons and target cells, resulting in the formation of functional synapses.^24^ Inspired by this, we explored whether Sema3C secreted by HCC cells interacted with other TME components to reshape the tumor niche, promoting HCC stemness. Gene Ontology (GO) pathway enrichment analysis of proteins bound to Sema3C and NRP1 revealed significant enrichment in extracellular matrix organization, integrin-mediated signaling pathway, and collagen fibril organization. These findings suggested that Sema3C may regulate the ECM in the HCC microenvironment (Fig. 5a). To assess the effect of Sema3C on the ECM remodeling, a collagen contraction assay was conducted. We found that after being treated with the supernatants of OE-Sema3C-MHCC-97L cells, the gel contraction of collagen I was significantly enhanced (Fig. 5b). The MMP and lysine oxidase (LOX) family proteins are responsible for collagen remodeling in cancer.^25,26^ Overexpression of Sema3C markedly increased *MMP2, MMP9, LOX*, and *LOXL2* levels, and treatment of MHCC-97L cells with rhSema3C elevated *MMP2, LOX*, and *LOXL2* but not *MMP9* (Supplementary Fig. 5a, b). To investigate the role of Sema3C in ECM remodeling in vivo, we performed orthotopic implantation of OE-Sema3C-MHCC-97L cells or control cells in nude mice. Tumors in mice injected with OE-Sema3C-MHCC-97L cells were significantly larger than those in control mice. Masson’s trichrome and PicroSirius red staining showed greater collagen fiber infiltration in OE-Sema3C tumors. IHC and quantitative analysis revealed increased α-SMA and collagen I expression in OE-Sema3C tumors compared to controls (Fig. 5c, d). These findings above revealed that Sema3C in HCC cells could exacerbate ECM deposition in the TME.

In addition to directly regulating ECM, considering the secretory properties of Sema3C, we explored whether HCC-derived Sema3C could transform HSCs into CAFs in a paracrine way, thereby promoting ECM production. First, we treated LX-2 cells with rhSema3C and observed morphological changes using IF. The results showed that rhSema3C treatment markedly increased the cytoplasmic volume of LX-2 cells and induced the formation of more cellular projections (Supplementary Fig. 5c). In addition, rhSema3C could also enhance the proliferation ability of LX-2 cells (Supplementary Fig. 5d).

We collected conditioned medium (CM) from HCC cells with varying Sema3C expression levels and treated LX-2 cells for 48 h. The CM from OE-Sema3C-HCC cells or rhSema3C significantly increased the mRNA levels of *ACTA2, COL1A1, ELN*, and *TGF-B1* (Supplementary Fig. 5e, f), as well as the protein expression of α-SMA and collagen I in LX-2 cells (Fig. 5e, f). Additionally, ELISA assay confirmed that Sema3C secreted by HCC cells promoted TGF-β1 production in LX-2 cells (Fig. 5g). Then the effect of Sema3C on the biological function of HSCs was verified. We found that CM from OE-Sema3C-HCC cells or rhSema3C significantly increased the capacity of LX-2 cells to chemoresistance, migration, and invasion as compared to controls (Fig. 5h, i). To verify the communication between HSCs and HCC cells, we collected CM from OE-Sema3C-activated LX-2 cells and co-cultured it with Hep3B cells. The results revealed that activated LX-2 upregulated stemness-related genes and enhanced chemoresistance in Hep3B cells, indicating that activated LX-2 cells were sufficient to induce stemness maintenance in HCC cells (Supplementary Fig. 5g, h).

To explore the impact of Sema3C-mediated interactions between HCC cells and HSCs on tumorigenesis in vivo, we constructed a xenograft HCC model by injecting HCC cells and HSCs subcutaneously into nude mice (Fig. 5j). Animals co-injected with OE-Sema3C Hep3B cells and LX-2 cells had a greater tumor weight and volume than all other groups (Fig. 5k, l). It is therefore suggested that HCC cells-derived Sema3C is involved in both remodeling ECM and activation of HSCs to form a supportive niche in TME.

### IL-6 and cholesterol biosynthesis are responsible for Sema3C-mediated HSCs activation

**Fig. 6:**
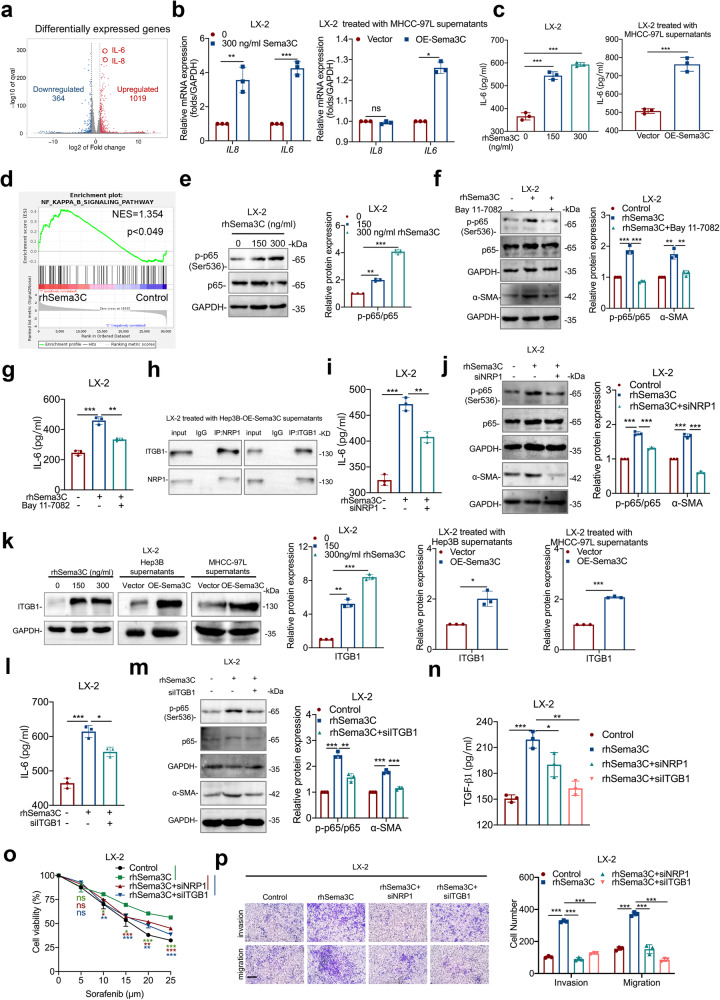
IL-6 and cholesterol biosynthesis are responsible for Sema3C-mediated HSCs activation. **a** The volcano map showed the differentially expressed genes of LX-2 cells treated with rhSema3C or PBS in vitro. HSCs were treated with rhSema3C or supernatants of MHCC-97L cells with different Sema3C expression levels. mRNA levels of *IL6* and *IL8* were detected using qRT-PCR (**b**). ELISA was used to examine the IL6 secretion level (**c**). **d** GSEA identified an enrichment of genes involved in the NF-κB pathway in rhSema3C-treated LX-2 cells. **e** Western blotting showed phosphorylated and total p65 protein expression levels in LX-2 cells treated with rhSema3C. LX-2 cells were pre-treated with Bay 11-7082 and subsequently stimulated with rhSema3C, the levels of phosphorylated p65, total p65, and α-SMA expression were determined by western blotting (**f**), and IL6 secretion levels were examined by ELISA assay (**g**). **h** LX-2 cells were treated with OE-Sema3C-Hep3B cell-derived supernatants, Co-IP assay was used to detect the interaction between ITGB1 and NRP1. LX-2 cells were transfected with siNRP1 and subsequently stimulated with rhSema3C, IL6 secretion levels were detected by ELISA (**i**). The phosphorylated p65, total p65, α-SMA, and HMGCR expression levels were determined by using western blotting (**j**). **k** LX-2 cells were treated with a dosage of rhSema3C or supernatants of Sema3C-overexpressed HCC cells, and ITGB1 protein expression was determined by western blotting. LX-2 cells were transfected with siITGB1 and subsequently stimulated with rhSema3C, and the IL6 secretion levels were detected by ELISA assay (**l**), the phosphorylated p65, total p65, and α-SMA expression levels were determined by using Western blotting (**m**). LX-2 cells were treated with siNRP1, siITGB1 or rhSema3C as indicated, the secretion of TGF-β1 in each group was detected by ELISA (**n**), the chemotherapy resistance of LX-2 cells was evaluated by MTT assay (**o**), and migration and invasion were performed by Transwell assay, scale bar, 200 μm (**p**). rhSema3C recombinant human Sema3C; Data are presented as means ± SD. ns not significantly; *P < 0.05, **P < 0.01, and ***P < 0.001

According to the above findings, we explored the potential mechanism by which Sema3C induces HSCs activation. Thus, an RNA sequencing assay was performed based on LX-2 cells using rhSema3C or its control treatment. We identified 1383 differentially expressed genes (DEGs), with 1019 upregulated and 364 downregulated. *IL6* and *IL8* were the most significantly upregulated in the rhSema3C-treated groups compared to controls (Fig. 6a). Meanwhile, analysis of TCGA databases revealed a strong positive correlation between Sema3C expression and IL6, IL8 (CXCL8) expression in HCC (Supplementary Fig. 6a, b). To determine whether Sema3C directly promoted IL6 and IL8 production, we treated LX-2 cells with rhSema3C or supernatants from OE-Sema3C HCC cells, and only *IL6* mRNA level was significantly upregulated (Fig. 6b). Moreover, ELISA experiments confirmed that gradient doses of rhSema3C or supernatants of OE-Sema3C-MHCC-97L cells increased IL6 secretion in LX-2 cells (Fig. 6c). To further investigate the biological processes involved in Sema3C-regulated HSCs activation, enrichment analysis of DEGs from transcriptome sequencing was performed. We found that Sema3C not only positively regulated IL6 production but also influenced cholesterol biosynthesis and metabolic processes in HSCs (Supplementary Fig. 6c-e). Consistently, rhSema3C significantly increased the total cholesterol content in LX-2 cells in vitro (Supplementary Fig. 6f). We identified the major DEG involved in cholesterol biosynthesis and metabolism, finding that *HMGCS1, INSIG1, FASN*, and *HMGCR* expression levels were significantly increased in the rhSema3C-treated groups (Supplementary Fig. 6g). Considering that HMGCR is the rate-limiting enzyme in the mevalonate pathway for cholesterol biosynthesis and its high expression in various tumor tissues promoted tumor progression.^27,28^ Therefore, we explored whether Sema3C affected cholesterol metabolism mainly by regulating HMGCR expression in HSCs. The results showed that rhSema3C treatment upregulated HMGCR mRNA and protein levels in LX-2 cells (Supplementary Fig. 6h, i). To elucidate the pathway by which Sema3C promoted IL6 secretion and cholesterol synthesis, we first investigated whether the PI3K/AKT pathway, known to be activated in HCC cells, was also involved in regulating LX-2 cells. However, the results suggested that rhSema3C could not increase AKT phosphorylation in LX-2 cells (Supplementary Fig. 6j). GSEA analysis revealed significant enrichment of the NF-κB pathway in rhSema3C-treated LX-2 cells (Fig. 6d). Treatment with rhSema3C or supernatants from OE-Sema3C HCC cells increased NF-κB (p65) phosphorylation in LX-2 cells (Fig. 6e, Supplementary Fig. 6k). To verify the involvement of NF-κB in Sema3C-induced HSCs activation, we pre-treated LX-2 cells with the NF-κB inhibitor-Bay 11-7082 and found that subsequent rhSema3C stimulation did not increase α-SMA or HMGCR expression (Fig. 6f, Supplementary Fig. 6l), and it also antagonized IL6 secretion and cholesterol synthesis (Fig. 6g, Supplementary Fig. 6m). Next, to explore whether Sema3C promotes HSCs activation via NRP1 and ITGB1, we performed Co-IP experiments showing that CM from Sema3C-expressing Hep3B cells induced NRP1-ITGB1 interaction in LX-2 cells (Fig. 6h). Silencing NRP1 in LX-2 cells using siRNA abrogated rhSema3C-induced IL6 secretion, NF-κB phosphorylation, α-SMA and HMGCR expression, and cholesterol synthesis (Fig. 6i, j, Supplementary Fig. 6n-p). To identify the involvement of ITGB1 in Sema3C-mediated HSCs activation, we found that ITGB1 protein levels were significantly increased in LX-2 cells treated with rhSema3C or supernatants of OE-Sema3C-HCC cells (Fig. 6k). Knockdown of ITGB1 similarly antagonized Sema3C-induced IL6 secretion, NF-κB phosphorylation, α-SMA and HMGCR expression, and cholesterol synthesis (Fig. 6l, m, Supplementary Fig. 6q-s). Additionally, phenotypic experiments demonstrated that knocking down NRP1 or ITGB1 reduced rhSema3C-induced TGF-β1 secretion, as well as proliferation, invasion, and migration of LX-2 cells (Fig. 6n–p). These findings suggested that Sema3C promoted HSCs activation by upregulating IL6 secretion and cholesterol synthesis through NRP1 and ITGB1.

### CAFs-derived TGF-β1 up-regulates Sema3C expression via AP1 in HCC cells

**Fig. 7:**
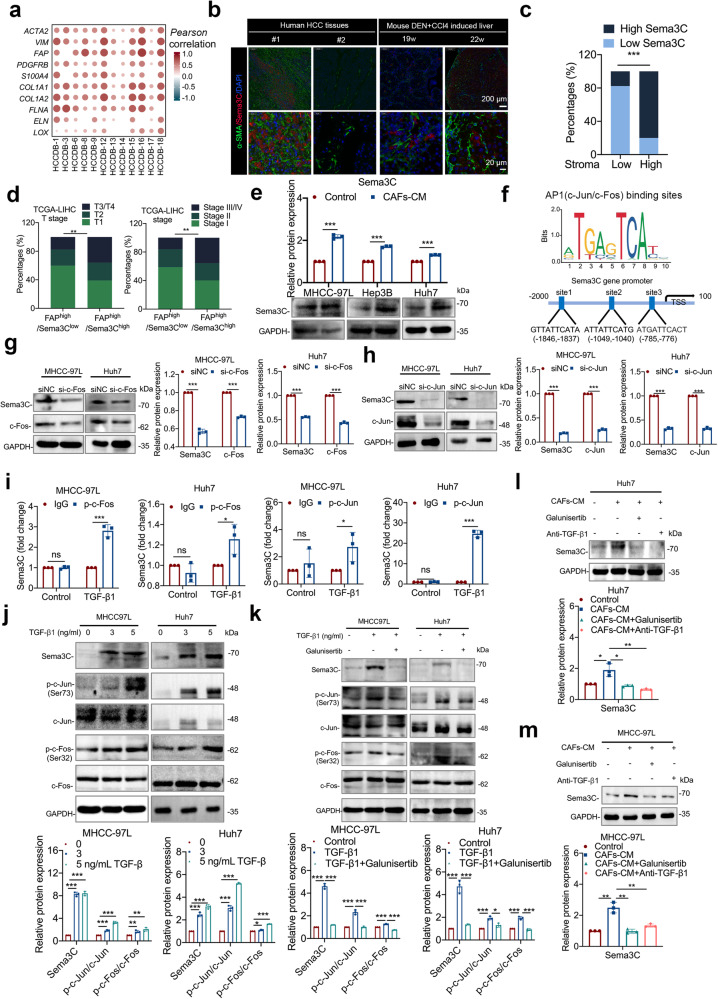
Sema3C expression is closely correlated with CAFs in HCC and is mediated by CAFs-derived TGF-β1. **a** Correlation between Sema3C expression and CAFs-associated genes (*ACTA2, VIM, FAP, PDGFRB*, and *S100A4*) and collagen-associated genes (*COL1A1, COL1A2, FLNA, ELN*, and *LOX*) in HCC using the HCCDB datasets. The dots in the figure indicate p < 0.05. The color intensity and size of the circle are proportional to the correlation coefficients. **b** Representative images of IF staining for low or high Sema3C expression (red) with different degrees of stromal infiltration (α-SMA-green) in HCC tissues or DEN+CCl_4_-induced mouse liver tissues. Cell nuclei were stained with DAPI (blue). **c** The correlation analysis revealed a positive correlation between high Sema3C expression and the stromal percentage in patients with HCC (total n = 27). **d** TCGA-LIHC analysis of the correlation between patients with T stage or stage and FAP^high^/Sema3C^low^ or FAP^high^/Sema3C^high^ in HCC. **e** Western blotting showed that treatment with CAFs-conditioned media (CAFs-CM) significantly increased Sema3C expression compared with the control in HCC cells. **f** AP1 (including c-Jun/c-Fos) transcriptional binding sites in the Sema3C gene promoter. **g**, **h** MHCC-97L and Huh7 cells were transfected with si-c-Fos or si-c-Jun as indicated and the levels of Sema3C, c-Fos, and c-Jun were validated by western blotting. **i** ChIP-qPCR analysis showed that TGF-β1 stimulation facilitated the binding of phosphorylated c-Jun (p-c-Jun) and phosphorylated c-Fos (p-c-Fos) to the Sema3C gene promoter. **j** The levels of Sema3C, phosphorylated c-Jun (p-c-Jun), total c-Jun, p-c-Fos, and total c-Fos in MHCC-97L and Huh7 cells treated with different concentrations of TGF-β1 were determined by using western blotting. **k** Western blotting indicated that TGF-β1-induced Sema3C expression and AP1 signal activation could be inhibited by galunisertib (TGF-β1 receptor type I Inhibitor). **l**, **m** Western blotting confirmed that galunisertib and the TGF-β1 neutralizing antibody (anti-TGF-β1) inhibited the Sema3C upregulation stimulated by treatment with CAFs-CM. Data are presented as means ± SD. *p < 0.05, **p < 0.01, ***p < 0.001

Research has shown that HSCs were the main source of CAFs in the TME, and CAFs could cross-talk with tumor cells to influence tumor progression in a paracrine way.^7^ Thus, we wondered whether Sema3C-induced CAFs activation could in turn regulate Sema3C expression in HCC cells to promote stemness maintenance, thereby creating a vicious cycle between stromal cells and tumor cells. First, analysis of the HCCDB database revealed a positive correlation between Sema3C expression and markers associated with CAFs, including *ACTA2, VIM, FAP, PDGFRB, S100A4*, and collagen-associated molecules, including *COL1A1, COL1A2, FLNA, ELN*, and *LOX* (Fig. 7a). We also found Sema3C to be significantly associated with the stromal score as calculated by using the R package “ESTIMATE” (Supplementary Fig. 7a). Furthermore, analysis using various algorithms in the TIMER2.0 database indicated a positive correlation between Sema3C expression and CAFs infiltration in HCC (Supplementary Fig. 7b). Immunofluorescence was employed to examine the spatial relationship between Sema3C expression and α-SMA^+^ CAFs in both human HCC samples and a mouse model of HCC induced by DEN+CCl_4_. We found that Sema3C expression was also increased in samples with high abundance of α-SMA^+^ CAFs infiltration, and the proximity of Sema3C to CAFs also indicated that Sema3C in HCC cells were constantly communicating with CAFs directly or indirectly (Fig. 7b, c). Furthermore, analysis of the TCGA-LIHC database revealed that HCC patients with high Sema3C expression and the presence of FAP^+^ CAFs subset showed a significant association with advanced tumor stages/T stages compared to those with low Sema3C expression and FAP^+^ CAFs. This suggested a clinical relevance of the interaction between Sema3C-expressing HCC cells and FAP^+^ CAFs in in driving malignant progression of HCC (Fig. 7d).

To verify the dynamic communication between HCC cells and CAFs, primary CAFs were isolated from HCC clinical samples (Supplementary Fig. 7c). Then, HCC cells were cocultured with the CM of CAFs in vitro to confirm this hypothesis. The results indicated that treatment with CAFs-derived CM increased Sema3C expression in HCC cells compared to the control (Fig. 7e, Supplementary Fig. 7d). Previous studies have shown that TGF-β1 secreted by activated CAFs was involved in the regulation of tumor cells,^29^ and our above KEGG enrichment analysis also showed that Sema3C might be related to the TGF-β1 signaling pathway (Fig. 3A). We investigated whether TGF-β1 in CAF supernatants could enhance Sema3C expression in HCC cells. Analysis of the TCGA database showed a positive correlation between Sema3C and TGF-β1 expression in HCC (Supplementary Fig. 7e). Moreover, treatment of HCC cells with gradients of TGF-β1 in vitro significantly upregulated Sema3C mRNA levels (Supplementary Fig. 7f).

We explored the mechanism of TGF-β1-mediated Sema3C upregulation in HCC cells by analyzing the transcription factor binding sites in the Sema3C gene promoter region. Three AP1 binding sites were identified upstream (−1846 to −776 nucleotides) of the transcription start site (TSS) (Fig. 7f). Comparative analysis of AP1 family gene expression in MHCC-97L and Huh7 cells revealed that *c-Jun* and *c-Fos* exhibited the highest expression levels among the family genes (Supplementary Fig. 7g). As expected, silencing c-Jun/c-Fos with siRNA significantly inhibited Sema3C expression in HCC cells (Fig. 7g, h). Through ChIP-qPCR analysis, it was observed that stimulation with TGF-β1 led to a notable increase in the binding of phosphorylated c-Jun (p-c-Jun) and phosphorylated c-Fos (p-c-Fos) to the Sema3C promoter (Fig. 7i). Furthermore, the levels of p-c-Jun and p-c-Fos were increased with a dosage TGF-β1 treatment (Fig. 7j). To verify the regulatory effect of the TGF-β1-AP1 signaling axis on Sema3C, we performed rescue experiments using galunisertib (an inhibitor of TGF-β receptor 1), or a TGF-β1–neutralizing antibody. The results showed that galunisertib could block TGF-β1-induced Sema3C expression and phosphorylation of c-Jun and c-Fos (Fig. 7k). Besides, both galunisertib and TGF-β1–neutralizing antibody could antagonize CAFs-CM-mediated Sema3C expression (Fig. 7l, m). Collectively, AP1 signaling was accountable for the TGF-β1–mediated Sema3C upregulation in HCC cells.

### Sema3C inhibition enhances sorafenib efficacy in HCC mouse model

**Fig. 8:**
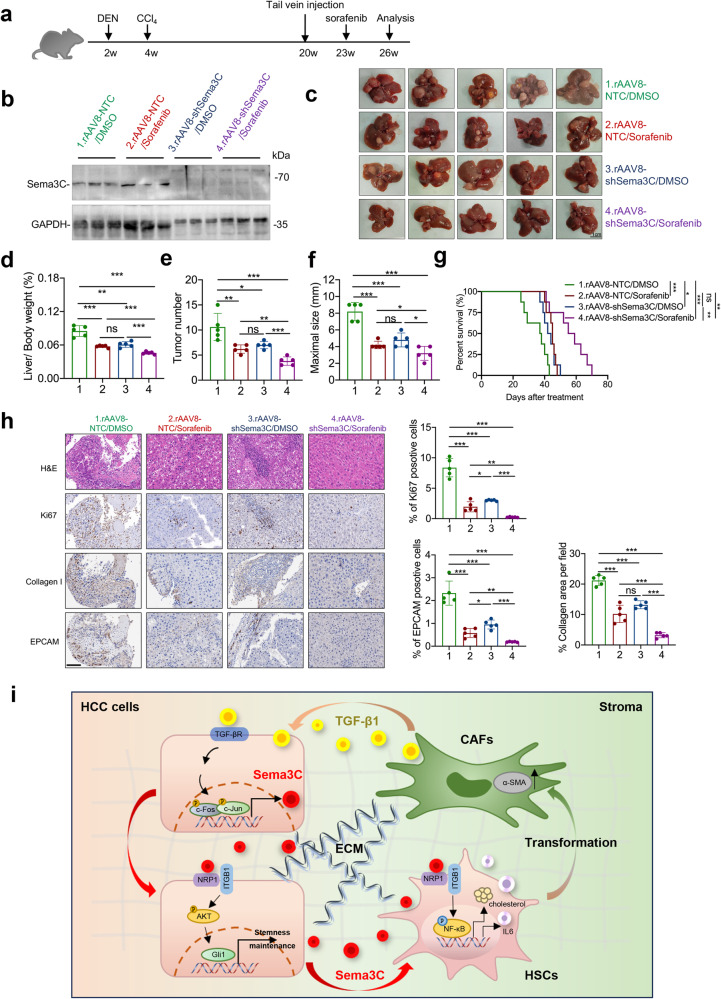
Sema3C inhibition enhances sorafenib efficacy in HCC mouse model. **a** Diagram demonstrated the time point of DEN+CCl_4_-induced mouse HCC model and treatment mode. **b** The mouse livers from the indicated groups were removed after 26 weeks, and the protein expression of Sema3C was detected by western blotting. **c** Representative images of mouse liver tissue from the indicated groups at 26 weeks (n = 5 per group), scale bar, 1 cm. Liver/body weight (**d**), tumor number (**e**), and maximal size of tumor of the indicated groups (**f**). **g** Kaplan–Meier survival curve showing percentage of overall survival of each annotated group (n = 8). **h** H&E, IHC staining and statistical analysis for Ki67, Collagen I, and EpCAM in HCC tumors harvested from the 4 treatment groups (n = 5 per group). Scale bar, 100 μm. **i** Schematic diagram showing that Sema3C mediates the interaction between CSCs and stroma in HCC. H&E hematoxylin-eosin, IHC immunohistochemistry; Data are presented as means ± SD. ns, not significantly; *p < 0.05, **p < 0.01, ***p < 0.001

The above studies have identified that Sema3C was up-regulated in sorafenib-resistant HCC cells and this elevated expression of Sema3C led to sorafenib resistance in vitro. However, targeted inhibitors for Sema3C are not commercially available nowadays. Therefore, we examined the therapeutic targeting of Sema3C by intravenous injection of rAAV8-shSema3C alone or in combination with sorafenib in a DEN+CCl_4_-induced HCC mouse model. At 20 weeks, the mice were injected with the rAAV8-shSema3C or its control rAAV8-shNTC via the tail vein. Three weeks later, the mice were segregated into 4 groups and then administrated with sorafenib at 30 mg/kg or DMSO control for 3 weeks (Fig. 8a). We randomly selected 3 mice from each group to verify the knockdown efficiency of Sema3C after removing liver tumors (Fig. 8b). We found that the combination of rAAV8-shSema3C and sorafenib significantly inhibited liver-to-body weight, tumor numbers, and the maximum size of tumors (Fig. 8c–f). Notably, The combination treatment of rAAV8-shSema3C and sorafenib significantly improved survival compared to mice treated with DMSO control, sorafenib alone, or rAAV8-shSema3C alone (Fig. 8g). IHC analysis of proliferative marker Ki67, Collagen I, and EPCAM in the excised livers from the four treatment groups revealed a marked decrease in the rAAV8-shSema3C/sorafenib combination group, suggesting knocked down of Sema3C did effectively impair proliferative capacity, stromal deposition, and stemness niche of the tumor (Fig. 8h). Collectively, these results suggested that inhibition of Sema3C sensitized HCC cells to sorafenib and synergically reduced tumor growth.

## Discussion

It is well known that 90% of HCC cases arise in the context of fibrosis or cirrhosis.^30^ During the progression from fibrosis to liver cancer, HSCs are continuously activated to produce a large amount of ECM.^31^ In HCC, activated HSCs are transformed into CAFs, constituting the main component of tumor stroma, often referred to as the “soil” of the tumor.^32^ Previous studies have mainly concentrated on how CAFs activate and support CSCs,^33^ but how CSCs, as the “seed” of tumor initiation, educate the tumor stroma has not been well studied. Our investigation revealed upregulated expression of Sema3C in both fibrotic liver, HCC tissues and peripheral blood HCC patients. Elevated Sema3C levels facilitated the activation of the AKT/GLi1/c-Myc signaling axis via binding to HCC cell surface receptors, NRP1 and ITGB1, in an autocrine manner, thus promoting HCC stemness. Additionally, Sema3C bound to NRP1 and ITGB1 in HSCs, activating downstream NF-kB signaling, which stimulated pro-inflammatory cytokine IL-6 production and enhanced HSCs cholesterol synthesis by upregulating HMGCR expression, synergistically driving HSCs transformation. Moreover, activated CAFs secreted TGF-β1 to activate downstream AP1 phosphorylation, thereby mediating Sema3C transcription and promoting its upregulation in HCC cells. Consequently, our discovery of Sema3C as a novel biomarker highlights its key role in mediating bidirectional communication between CSCs and the tumor stroma, driving a vicious cycle that accelerates HCC progression (Fig. 8i).

To identify molecules associated with stemness in HCC development, we initially explored proteins that are upregulated in both cirrhotic and HCC tissues as well as sorafenib-resistant HCC xenografts, and we focused on secreted proteins that may be involved in intercellular communication. Among the 13 molecules screened, we identified Sema3C, a secreted protein in the axon guidance family, which promotes CSC survival in glioblastoma and correlates with poor prognosis in various tumors.^19,34^ Sema3C is highly expressed and significantly correlated with tumor size, portal vein embolization, and distant metastasis in HCC.^15^ However, its role in tumor stemness and specific mechanisms remain unclear. Our study found that Sema3C was upregulated in cirrhosis and HCC tissues in a DEN+CCl_4_-induced mouse model and highly expressed in sorafenib-resistant HCC cells. According to the CSCs theory, CSCs are cancer cell subpopulations with high self-renewal capacity, therapeutic resistance, and tumor initiation.^35^ We revealed that Sema3C promoted stemness maintenance and tumorigenesis in HCC, consistent with its role in prostate, pancreatic, gastric cancers, and glioblastoma.^19,36–38^

TCGA analysis showed that patients with high Sema3C expression had significant enrichment in the PI3K/AKT, Wnt, Hippo, and Hedgehog pathways, all of which regulate CSCs biological activity.^12^ By manipulating Sema3C expression, we found that Sema3C only activates the AKT and Hedgehog signaling pathways, but not the Wnt and Hippo signaling pathways. Studies have shown that hyperactivation of AKT signaling promotes CSCs-like characteristics in HCC.^39–41^ A recent study revealed that Gli1 is highly expressed in HCC tissues, and hedgehog activation drives CSCs self-renewal.^42^ In addition, c-Myc, a downstream target of AKT and Hedgehog pathways, exerts a crucial role in hepatocarcinogenesis.^43,44^ Considering that CSC activity is regulated by multiple interconnected signaling pathways, these pathways collectively promote the proliferation of CSCs.^12^ Previous studies have demonstrated that PI3K/AKT activity is essential for Gli1-dependent Hedgehog signaling in response to IGF-1 stimulation in esophageal cancer and renal cell carcinoma.^18,45^ Accordingly, we explored whether Sema3C is a novel stimulator to mediate the crosstalk between AKT and the Hedgehog signaling pathway to regulate the stemness in HCC. We found that inhibiting AKT with MK-2206 attenuated Sema3C-mediated stemness and reduced Gli1 and c-Myc expression, providing new evidence that Sema3C functions via the AKT/Gli1/c-Myc axis in HCC cells.

In general, as a secreted protein, Sema3C needs to bind to receptors on the cell surface to perform specific functions. Sema3C has two directly binding receptors, NRP1 and NRP2, whose binding affinities are similar.^16^ NRP1 is highly expressed in HCC, contributing to tumor growth, metastasis, vascular remodeling, recurrence, and increased liver CSC populations in HCC patients.^20,21^ We demonstrated that Sema3C drives stemness maintenance by binding to NRP1. NRP1 has multiple binding partners, including plexins, integrins, EGFR, and VEGFR et al.^16^. By using co-immunoprecipitation and mass spectrometry, we screened that only ITGB1 in the integrin family binds to Sema3C and NRP1. ITGB1, a major integrin subfamily member, is a cell surface receptor involved in various biological processes.^46^ It is aberrantly expressed in many tumors, including HCC, where it promotes tumor progression by regulating the cell cycle.^22^ Our study showed that inhibition of ITGB1 also antagonized Sema3C-mediated stemness characteristics, and signatures constructed with ITGB1, NRP1, and Sema3C expression also suggested that high signatures had the worst prognosis in HCC patients. However, this does not rule out that Sema3C acts by binding to other receptors, and we here showed that ITGB1 may be the main receptor mediating Sema3C on stemness, which also indicated that the function of Sema3C is context-dependent.

Several studies have reported that Semaphorins interact with surrounding microenvironment to promote tumor progression.^47^ However, whether Sema3C is involved in stromal remodeling has not been investigated. Our findings demonstrate for the first time that Sema3C reshapes the tumor stroma to maintain tumor stemness. The dynamic interaction between cancer cells and the ECM is crucial for regulating tumor growth.^48^ Tumor cells can remodel the ECM by regulating MMP and LOX family proteins.^25,26^ We found that Sema3C increases MMP2/9, LOX, and LOXL2 expression, enhances collagen I contraction in vitro, and promotes collagen deposition in an orthotopic HCC mouse model. Previous studies have shown that integrins interact with ECM proteins to induce cancer stemness and drug resistance through FAK and AKT signaling activation.^49^. Sema3C may also play a regulatory role in ECM by activating AKT signaling through the ITGB1 receptor. Since ECM is largely produced by CAFs in the TME, we explored the role of secretory Sema3C on HSCs, a major source of CAFs in HCC.^7^ A previous study highlighted elevated Sema3C expression in liver fibrosis and HSCs, suggesting its role in HSC activation and fibrosis progression.^50^ However, our research focused on HCC progression, observing Sema3C expression in HCC tissues and cells. Sema3C facilitated HSCs activation in a paracrine manner, mediating intercellular communication within the HCC microenvironment. Moreover, we identified distinct mechanisms underlying Sema3C-mediated activation of HSCs within the TME compared to liver fibrosis.

Transcriptome sequencing analysis of HSCs treated with rhSema3C revealed a significant association between Sema3C and IL8 and IL6. Further validation using Sema3C-manipulated HCC cell supernatants demonstrated a remarkable induction of IL6 production in HSCs. Mounting evidence suggests that increased IL6, an inflammatory cytokine, promotes HCC progression and HSC activation, but the detailed mechanism remains elusive.^51–53^ Our enrichment analysis revealed Sema3C regulated cholesterol synthesis in HSCs. Previous research has shown that increased free cholesterol in HSCs sensitizes them to TGF-β1-induced activation.^54^ HMGCR, a key enzyme in cholesterol biosynthesis, promotes HCC growth.^55^ Our study demonstrates that Sema3C upregulates HMGCR expression in HSCs to enhance cholesterol synthesis. GSEA analysis suggested NF-κB was involved in Sema3C-induced HSC activation. Bay11-7082, an NF-κB inhibitor, antagonized IL-6 production, α-SMA and HMGCR expression, and cholesterol synthesis induced by Sema3C. Additionally, we found that Sema3C activation of HSCs depends on NRP1 and ITGB1 binding.

Recent findings indicated that CAFs serve as a significant source of cytokines and chemokines that drive cancer stemness.^7^ We found that Sema3C expression in HCC cells is positively correlated with CAFs infiltration abundance. Moreover, the distribution of Sema3C-positive HCC cells and CAFs are spatially intertwined in both human HCC tissues and murine-induced HCC models. In vitro co-culture experiments demonstrated that Sema3C expression in HCC cells was elevated upon exposure to supernatant extracted from CAFs. Previous studies have shown that TGF-β1 secretion levels are comparable in HCC cells and CAFs,^56^ and that Sema3C can be induced by TGF-β1 in lung cancer.^57^ We also confirmed that CAFs-derived TGF-β1 promotes Sema3C expression in HCC cells, a process attenuated by TGF-β1 neutralizing antibodies and TGFBR inhibitors. Mechanistic insights further revealed that TGF-β1-activated AP1 directly interacted with the Sema3C promoter region in HCC cells. Prior research demonstrated that TGF-β promoted HCC growth, invasion, immune evasion, and ECM deposition.^58^ Galunisertib, a TGF-β inhibitor, showed promise in slowing advanced HCC development.^59^ Combining TGF-β targeting drugs in the future could inhibit HCC proliferation and immune evasion by reducing TGF-β expression, and concurrently down-regulate Sema3C expression to enhance efficacy in reducing drug resistance and preventing tumor recurrence.

Accumulating evidence underscores the inadequacy of targeting tumor cells alone in achieving satisfactory therapeutic outcomes. Recognizing the interplay between the TME and tumor cells, particularly CSCs, offers a strategy to impede HCC progression. We explored targeting Sema3C, alone or with sorafenib, in an immunocompetent mouse model using intravenous rAAV8-shSema3C. The combination treatment effectively inhibited tumor growth, suggesting that Sema3C suppression sensitizes HCC to sorafenib.

Taken together, our study sheds light on the reciprocal interactions between the tumor stroma and CSCs, elucidating their pivotal role in tumorigenesis. Beyond receiving survival signals from the TME, CSCs play a direct role in reshaping the tumor stroma to create a conducive niche for tumor progression. We uncovered that Sema3C augments stemness through autocrine signaling, while simultaneously promoting the activation of HSCs through paracrine signaling. Additionally, CAFs within the stroma upregulate Sema3C expression via TGF-β1 secretion, thereby establishing a detrimental feedback loop between tumor and stromal cells, ultimately driving HCC progression. Our findings not only enhance our understanding of HCC pathogenesis but also underscore the clinical significance of disrupting the crosstalk between CSCs and the tumor stroma to combat HCC. In the future, different liver cancer models will be used to verify the role of Sema3C in diverse etiologies of HCC, such as the hydrokinetic tail vein (HTV) -NRasV12+Myr-AKT proto-oncogene driven HCC tumors and non-alcoholic steatohepatitis (NASH)-HCC mouse models. Furthermore, we are now actively developing small molecule inhibitors targeting Sema3C and utilizing tumor organoids for drug screening, thereby enhancing drug development efficiency.

## Materials and methods

### Cell lines and cell culture

Liver cancer cell line Hep3B were purchased from Procell (Wuhan, China), liver cancer cell lines (Huh7, MHCC-97L, HepG2 and SMMC7721) and the human HSC line LX-2 were purchased from Immocell (Xiamen, China), Human hepatocytes MIHA cell line was obtained from Fenghui (Hunan, China), all liver cancer cell lines and LX-2 were cultured in DMEM (Gibco, United States) supplemented with 10% fetal bovine serum (FBS, Invitrogen, United States), MIHA cells were culture in RPMI 1640 (Gibco, United States) containing 10% FBS and 1% penicillin-streptomycin. All cells were incubated at 37 °C in humidified air with 5% CO_2_.

For CAFs isolation, fresh HCC tissues were cut into small pieces and digested in DMEM-F12 containing 1 mg/mL collagenase I and 100 U/mL hyaluronidase at 37 °C for 2 h. The digested mixture was then filtered through a 70 μm filter. The filtered cells were centrifuged and washed twice with PBS. Subsequently, the cells were re-suspended in DMEM-F12 medium supplemented with 10% FBS and 1% penicillin–streptomycin and transferred to culture flasks. The cultures were maintained at standard culture conditions. During the culture process, non-adherent cells were washed away, allowing CAFs to adhere to the flask and continue growing. The cultures were maintained for an additional 2–3 weeks to allow for CAF expansion.

### Collection of conditioned medium (CM)

CAFs or HCC cells were seeded in 6-well plates at a density of 1 × 10^5^ cells per well. After allowing the cells to adhere for 24 h, the medium was removed and replaced with fresh serum-free medium. The cells were then incubated for an additional 48 h. Following this incubation, the cell supernatant was collected and centrifuged at 10,000 *g* for 10 min at 4 °C to remove cell debris.

To verify the effect of TGF-β1 on Sema3C expression in HCC, 1 µg/mL anti-TGF-β1 antibody or an IgG control (Proteintech, China) was added to the CM of CAFs and incubated at room temperature for 1 h. The treated CM was then used to co-culture with the corresponding HCC cells.

### Clinical samples and public HCC cohorts

Serum samples were obtained by centrifugation from the peripheral blood of HCC patients before surgery and from healthy individuals. Primary HCC tissue samples were collected from patients following HCC resection at the Second Affiliated Hospital of Nanjing Medical University. Informed consent was obtained from all patients, and the study was approved by the Ethics Committee of the Second Affiliated Hospital of Nanjing Medical University ([2022]-KY-180-01). None of the HCC patients had received prior chemotherapy or radiotherapy before the operation.

The expression and clinical data of HCC were retrieved from the Cancer Genome Atlas (TCGA) database. Gene expression data for multiple HCC cohorts were downloaded from HCCDB (http://lifeome.net/database/hccdb/home.html). Microarray datasets (GSE14323, GSE5975, GSE6764, GSE14520, GSE36133 and GSE121153) were downloaded from the GEO (http://www.ncbi.nlm.nih.gov/geo/). Sema3C expression in HCC cell lines was analyzed using the GSE36133 dataset, which includes 10 non-differentiated and 5 differentiated HCC cell lines.^60^ Single-cell dataset analysis of liver cancer was conducted using data from TISCH2 (http://tisch.comp-genomics.org/search-gene/).

### Lentivirus production, small interfering RNA (siRNA), and cell transduction

The lentivirus overexpressing Sema3C was constructed and synthesized by miaolingbio. Inc (Wuhan, China). shRNA vector targeting Sema3C and ITGB1 were synthesized by shRNA-encoded DNA oligos and cloned into the pLKO.1 vector (Addgene). The target sequence used against Sema3C was as follows: 5′-CGTGTAATTCAGACTTTCAAT-3′. The target sequence used against ITGB1 was as follows: 5′-GCCTTGCATTACTGCTGATAT-3′.

siRNAs targeting Sema3C, NRP1, and NC were designed and synthesized by Ribobio (Guangzhou, China). siRNAs targeting c-Jun, c-Fos, and NC were synthesized by miaolingbio (Wuhan, China). siRNAs targeting ITGB1 and NC were synthesized by GENEray (Shanghai, China). Lipofectamine 2000 (Invitrogen) was used to transfect the corresponding siRNA into the cells according to the protocol. Cells were cultured subsequently for 72 h.

### RNA isolation, cDNA synthesis, and quantitative real-time RT-PCR (qRT-PCR)

For RNA extraction, 1 mL of TRIZOL reagent (ThermoFisher, USA) was added to 1 × 10^6^ cells and processed according to the manufacturer’s instructions. The RNA was extracted using the appropriate chemical reagents and the RNA pellet was re-suspended in enzyme-free water. cDNA was synthesized from 1 µg of RNA using HiScript III RT SuperMix (Vazyme, China). To compare relative gene expression between experimental groups, qRT-PCR was performed using SYBR Green (Vazyme, China). Genes was normalized to GAPDH by the 2^−ΔΔCt^ method. The primer sequences are listed in Supplementary Table S1.

### Western blotting

Cells were lysed using RIPA buffer supplemented with protease and phosphatase inhibitors (1 mM PMSF). The lysates were collected by centrifugation at 12,000 × *g* for 15 min at 4 °C to remove cell debris. Protein concentrations were determined using the BCA protein assay kit (Beyotime, China), according to the manufacturer’s instructions. An equal amount of protein (30 µg per sample) was separated on a 10% SDS-PAGE gel. Following electrophoresis, proteins were transferred to a PVDF membrane. After transfer, membranes were blocked with Tris-buffered saline (TBS) containing 0.1% Tween-20 (TBST) and 5% Bovine Serum Albumin (BSA) for 1 h at room temperature. The membranes were then incubated with the appropriate primary antibodies diluted in TBST containing 5% BSA overnight at 4 °C. After washing three times with TBST, the membranes were incubated with horseradish peroxidase (HRP)-conjugated secondary antibodies for 1 h at room temperature. The membranes were washed again three times with TBST. Finally, the protein bands were visualized using Enhanced Chemiluminescence (ECL) reagent (NCM Biotech Co., Ltd, China) and captured on a chemiluminescence imaging system. The specific antibodies and their respective suppliers are detailed in Supplementary Table S2.

### MTT assay, colony formation, apoptosis and ethynyl deoxyuridine (EdU) assays

Cells were seeded into 96-well plates at a density of 4000 cells per well and incubated with MTT reagent (Beyotime, China) following the manufacturer’s instructions. Absorbance was measured at 570 nm using a microplate reader to determine cell viability.

For colony formation assays, cells were plated at a density of 500 cells per well in 6-well plates and incubated for 2 weeks to allow colony formation. Colonies were subsequently fixed with 4% paraformaldehyde (PFA) for 15 min at room temperature and stained with 0.5% crystal violet solution (Beyotime, China) for 30 min. The number of colonies containing at least 50 cells was counted under a microscope.

Apoptosis was assessed using FITC-conjugated Annexin-V and Propidium Iodide (PI) staining (Vazyme, China). Cells were collected, washed twice with cold PBS, and resuspended in binding buffer. FITC-Annexin-V and PI were added, and the cells were incubated on ice in the dark for 30 min. Apoptotic cells were then analyzed by flow cytometry within 1 h of staining.

For the EdU incorporation assay, LX-2 cells were seeded in 96-well plates and incubated overnight. Cells were then labeled with EdU (Beyotime, China), followed by fixation, permeabilization, and staining. Images were captured from at least three random fields per well using a fluorescence microscope, and the percentage of EdU-positive cells was calculated.

### Sphere formation assay

HCC cells were seeded at density of 1000 cells/mL in 24-well ultra-low attachment plates (Corning) and cultured in DMEM-F12 medium (GIBCO). The medium was supplemented with B27 (1:50, Invitrogen), 20 ng/mL EGF (PeproTech), 20 ng/mL basic FGF (PeproTech), 1% penicillin/streptomycin, and 1% methylcellulose (Sigma-Aldrich). After 7–10 days of incubation, spheroids were counted.

### Transwell assay

For the in vitro migration assay, cells were resuspended in serum-free medium and placed in the upper chamber of a transwell apparatus with an 8-μm pore size membrane (Millipore). For the invasion assay, cells were seeded onto a Matrigel-coated transwell insert (Corning). The lower chamber was filled with DMEM containing 10% FBS to serve as a chemoattractant. After 24 or 48 h of incubation, cells on the upper surface of the membrane were removed, and the apparatus was fixed with 4% PFA and stained with crystal violet for 30 min. The migrated or invaded cells on the lower surface were counted under a microscope

### Collagen gel contraction assay

Collagen gel preparation was performed following previously described methods.^61^ A total of 7.5 × 10^4^ cells were resuspended in DMEM containing 10% FBS. The cell suspension was then mixed with 3 mg/mL rat-tail collagen I (Solarbio, China) to achieve a final concentration of 1 mg/mL collagen I. The mixture’s pH was adjusted to 7.4 using 0.1 M NaOH. Subsequently, 500 µL of this mixture was transferred into each well of a 24-well plate and allowed to solidify at room temperature for 30 min.

After solidification, the gels were treated with CM from MHCC-97L cells with different Sema3C expression levels as specified. The plates were then incubated at 37 °C in a humidified atmosphere with 5% CO_2_. After three days, the gels were gently detached from the edges of the wells. Gel contraction was monitored and quantified by measuring the gel area using ImageJ software.

### Enzyme-linked immunosorbent assay (ELISA)

Cell supernatants from different treatment groups were collected in the above manner. Secreted Sema3C levels were quantified using an ELISA kit from MEIMIAN. Additionally, secreted TGF-β1 and IL-6 were measured using ELISA kits from R&D Systems, following the corresponding protocols.

### Immunohistochemistry (IHC) staining

Human HCC samples, mouse HCC xenograft tissues, and liver tissues were embedded in paraffin and sectioned into 5 μm-thick slices. Slides underwent antigen retrieval in citrate buffer at 95 °C for 40 min, followed by blocking with 5% horse serum for 1 h. Primary antibodies were then applied overnight at 4 °C, including anti-Ki67 (1:100, Servicebio), anti-p-AKT (1:100, Cell Signaling Technology), anti-Gli1 (1:100, Cell Signaling Technology), anti-c-Myc (1:100, Proteintech), anti-Sema3C (1:100, Abmart), anti-α-SMA (1:100, Servicebio), and anti-Collagen I (1:100, Servicebio). After washing, sections were incubated with either anti-rabbit HRP or anti-mouse HRP-conjugated secondary antibodies.

### Immunofluorescence (IF) staining

For staining HCC cells, cells were first fixed with 4% PFA, permeabilized with 0.1% Triton X-100, and then blocked with 5% BSA. The cells were incubated with primary antibodies against α-SMA (1:100, Servicebio) or Collagen I (1:100, Servicebio), followed by incubation with FITC-conjugated goat anti-rabbit IgG (H + L) (1:200, Servicebio). After washing with PBS, the cells were stained with DAPI for 10 min and visualized using a fluorescence microscope.

For tissue staining, slides were blocked and incubated overnight at 4 °C with primary antibodies against α-SMA (1:100, Servicebio) or Sema3C (1:100, R&D Systems). The slides were then incubated with Alexa Fluor 594- or 488-conjugated secondary antibodies (Invitrogen). After washing with PBS, the slides were stained with DAPI. Images were captured using an OLYMPUS FV2000 fluorescence microscope.

### Chromatin immunoprecipitation (ChIP) assay

The ChIP assay was conducted using the Simple ChIP Enzymatic ChIP Kit (#9003, Cell Signaling Technology) following the protocol. In brief, cells were crosslinked with 1% formaldehyde to preserve protein–DNA interactions. The DNA was then sonicated into fragments and immunoprecipitated using antibodies against p-c-Jun (Ser73) (Cell Signaling Technology), p-c-Fos (Ser32) (Cell Signaling Technology), or a rabbit IgG control. The immunoprecipitation was performed at 4 °C overnight. The enrichment of specific DNA sequences in the Sema3C promoter region was analyzed by qRT-PCR. Primer sequences used for this analysis are provided in Supplementary Table S1.

### Co-immunoprecipitation (Co-IP) and mass spectrometry (MS) analysis

Immunoprecipitation assay was performed by rProtein A/G Magnetic IP/Co-IP Kit following the instructions (ACE biotechnology, China). Briefly, the cells were washed twice with cold PBS and lysed with cold-enhanced lysis/wash buffer supplemented with protease inhibitors on ice for 5 min. The cell lysates were then collected and incubated overnight at 4 °C with either anti-Flag or anti-HA antibodies, or the corresponding IgG control. The next day, rProtein A/G MagPoly Beads were prepared by washing with lysis/wash buffer (enhanced) three times and incubated with the lysate-antibody conjugate mixture with gentle shaking for 2 h. After incubation, the complex was centrifuged and washed gently with cold lysis/wash buffer (enhanced). Finally, the protein was then eluted with neutralization buffer containing 5X loading buffer for western blot analysis.

Mass spectrometry analysis: For solid gel samples, after decolorization and breaking treatment, the gel was immersed in 20 ng/µL Trypsin solution dissolved in 50 mM NH4HCO3, and enzymatically hydrolyzed at 37 °C for 16 h, followed by cold freezing and drying of peptide extract. Before the test, the peptide was detected by a Q Exactive TM HF-X mass spectrometer. The mass spectrometry proteomics data have been deposited to the ProteomeXchange Consortium (https://proteomecentral.proteomexchange.org) via the iProX partner repository,^62,63^ with the dataset identifier PXD052807.

### RNA-seq analysis

Total RNA extraction from LX-2 cells was isolated using the TRIzol reagent (thermofisher), and RNA were quality-controlled by NanoDrop ND-1000 (NanoDrop, USA). cDNA was synthesized from the fragmented RNA by using Invitrogen SuperScript^TM^ II Reverse Transcriptase (cat. 1896649, CA, USA). RNA sequencing was conducted utilizing the illumina NovaseqTM 6000 platform (LC Bio Technology, China). Subsequently, differential gene expression analysis was carried out employing the Cufflinks package and cuffmerge (fold change > 2 and p < 0.05). Bioinformatic analysis was executed using the OmicStudio tools accessible at https://www.omicstudio.cn/tool. Visual representations such as volcano plots were generated utilizing R programming language on the OmicStudio platform (https://www.omicstudio.cn/tool). The data will be available at https://www.ncbi.nlm.nih.gov/geo (GSE268894).

### Liver cancer mouse model

The hepatocarcinogenesis models were established following previously described protocols.^64^ In summary, male C57BL/6 mice received a single intraperitoneal injection of diethylnitrosamine (DEN, 25 mg/kg) at 2 weeks of age. Starting from 4 weeks of age, mice were intraperitoneally injected with carbon tetrachloride (CCl_4_, 5 ml/kg of a 3:1 mixture with olive oil) twice weekly until the specified endpoint. Tumor initiation was observed around 20 weeks of age, with the humane endpoint set at 26 weeks.

For combination therapy, recombinant adeno-associated virus serotype 8 (rAAV8)-shSema3C or rAAV8-shNC (at the dosage of 1 × 10^11^ vg/mouse) was delivered into mice intravenously in 100 μl PBS per mouse. The sequence of shRNA against mouse Sema3C is 5′-CGATGCTCTTTCAACCCGAAT-3′. After 3 weeks, mice were treated with sorafenib at 30 mg/kg for 3 weeks.

### In vivo tumorigenicity experiments

The experimental procedures were conducted in accordance with guidelines from the Institutional Animal Care and Use Committee of Southeast University Medical School (approval number: 20220714060). HCC cells were prepared in varying concentrations and suspended in a mixture of 50 μl serum-free medium and 50 μl Matrigel Matrix (BD Biosciences). These cell suspensions were subcutaneously injected into the flanks of five-week-old male BALB/c nude mice, with each mouse receiving two injections, one in each flank. Different groups of cells (shNC vs shSema3C; Vector vs OE-Sema3C) were injected into separate sets of mice. Tumors were collected at the end of the experiment for analysis. The frequency of tumor-initiating cells was determined using the Extreme Limiting Dilution Analysis (ELDA) software.^65^

To investigate the activation of HCC cell-derived Sema3C on HSCs and its impact on tumorigenesis in vivo, 5 × 10^5^ HCC cells, either alone or co-injected with LX-2 cells at a 1:1 ratio, were subcutaneously administered into the flanks of five-week-old male BALB/c nude mice, as previously described. Once tumors became palpable, their sizes were measured every 5 days using calipers. Tumor volumes were determined using the formula: volume (mm³) = L × W² × 0.5, where L is the longest diameter and W is the shortest diameter.

To assess the impact of Sema3C on ECM remodeling in vivo, we inoculated BALB/c nude mice with 1 × 10^6^ MHCC-97L cells expressing either empty vector (EV) or overexpressing Sema3C (OE-Sema3C) into the left hepatic lobe. After an 8-week duration, liver tissues were harvested for histological analyses including hematoxylin and eosin (H&E) staining, Masson’s trichrome staining, picrosirius red staining, and IHC.

### Statistical analysis

Statistical analyses were conducted using R software (version 4.1.2) and GraphPad Prism 8. All data are presented as mean ± standard deviation (SD). Group comparisons were performed using two-tailed Student’s *t* tests, while multiple comparisons were assessed using one-way ANOVA. Survival rates were evaluated using Kaplan-Meier curves with log-rank tests. Statistical significance was indicated as follows: ns, not significant; *p < 0.05; **p < 0.01; ***p < 0.001.

### Supplementary information

R3-Supplementary_Materials-revised

## Data Availability

Public data resources: TCGA-LIHC were obtained from GDC (https://portal.gdc.cancer.gov/). Gene expression data for multiple HCC cohorts were downloaded from HCCDB (http://lifeome.net/database/hccdb/home.html). Microarray datasets were downloaded from the GEO (http://www.ncbi.nlm.nih.gov/geo/). Immune infiltration data can be obtained from Sangerbox (http://sangerbox.com/home.html) and TIMER2.0 databases (http://timer.cistrome.org/). scRNA-seq is available at TISCH2 (http://tisch.comp-genomics.org/search-gene/). RNA-seq is available at https://www.ncbi.nlm.nih.gov/geo/query/acc.cgi?acc=GSE268894. The mass spectrometry proteomics data has been uploaded and is accessible at https://proteomecentral.proteomexchange.org, with the dataset identifier PXD052807.
