# Supplementary material for: Semaphorin 3C (Sema3C) reshapes stromal microenvironment to promote hepatocellular carcinoma progression
Source: Signal Transduct Target Ther. 2024 Jul 3;9:169. doi: 10.1038/s41392-024-01887-0 (PMC11220018; doi:10.1038/s41392-024-01887-0)
Supplement: Supplementary file 1 — R3-Supplementary_Materials-revised [file 41392_2024_1887_MOESM1_ESM.docx]

Supplementary Materials for

Sema3C reshapes stromal microenvironment to promote hepatocellular carcinoma progression

Hao Peng^1#^, Meng Yang^2#^, Kun Feng^3^, Qingpeng Lv^3^, Yewei Zhang^3, 1*^.

Correspondence to: zhangyewei@njmu.edu.cn

**This PDF file includes:**

Figures. S1 to S7

Tables S1 to S2


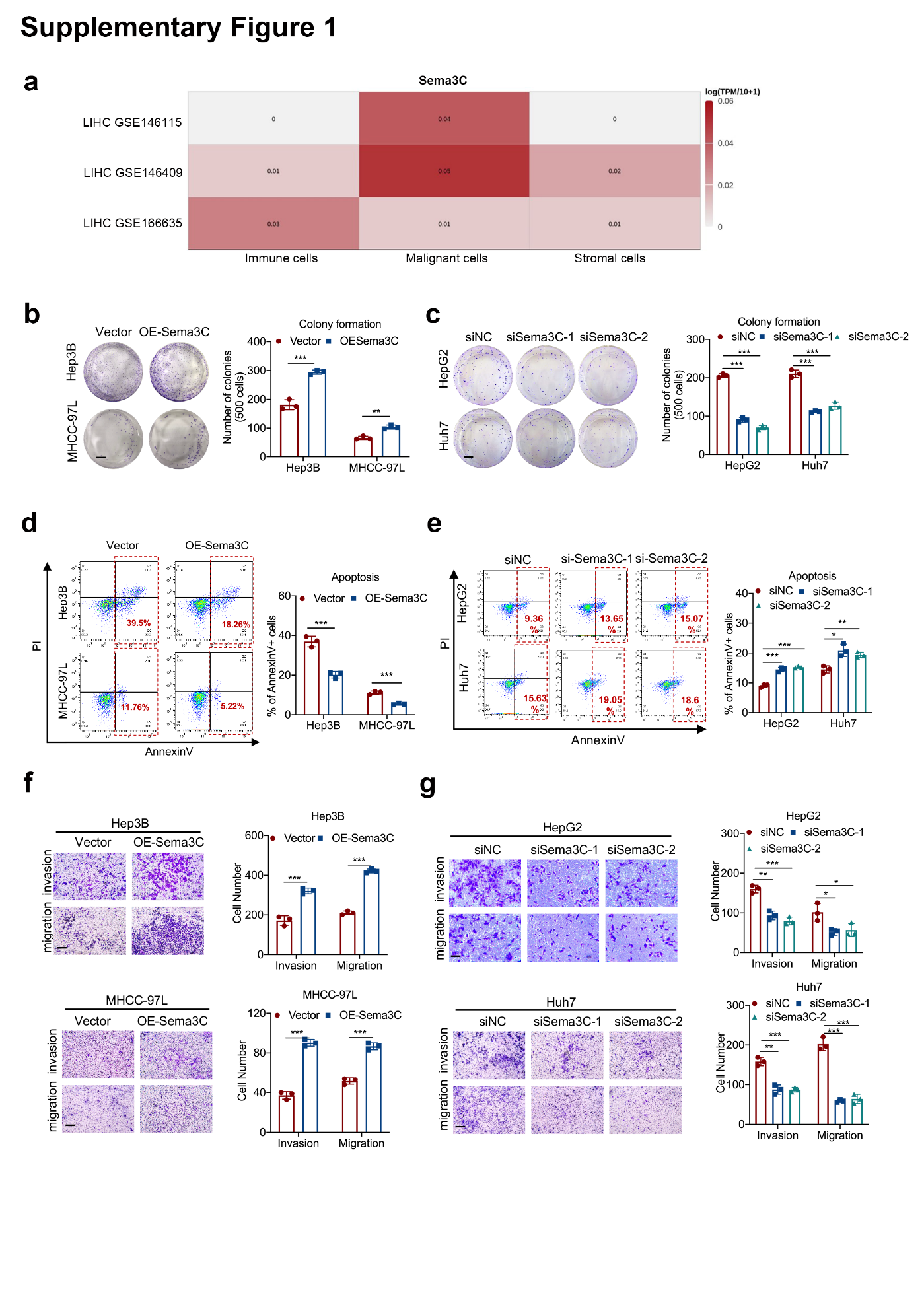


**Supplementary Figure S1. Effects of Sema3C on proliferation, apoptosis, invasion, and migration of HCC cells**

(a) Three different single-cell sequencing databases were used to analyze Sema3C expression in various cell types in HCC.

(b, c) The effect of Sema3C overexpression (b) and knockdown (c) on HCC cell proliferation was assessed by colony formation assay. Scale bar, 5 mm.

(d, e) The effect of Sema3C overexpression (D) and knockdown (E) on HCC cell apoptosis was assessed by flow cytometry.

(f, g) The effects of Sema3C overexpression (f) or knockdown (g) in HCC cells on migration and invasion were assessed by a transwell assay. Scale bar, 200 μm. Data are presented as means ± SD. ns, not significantly; *p <0.05, **p <0.01, ***p <0.001.


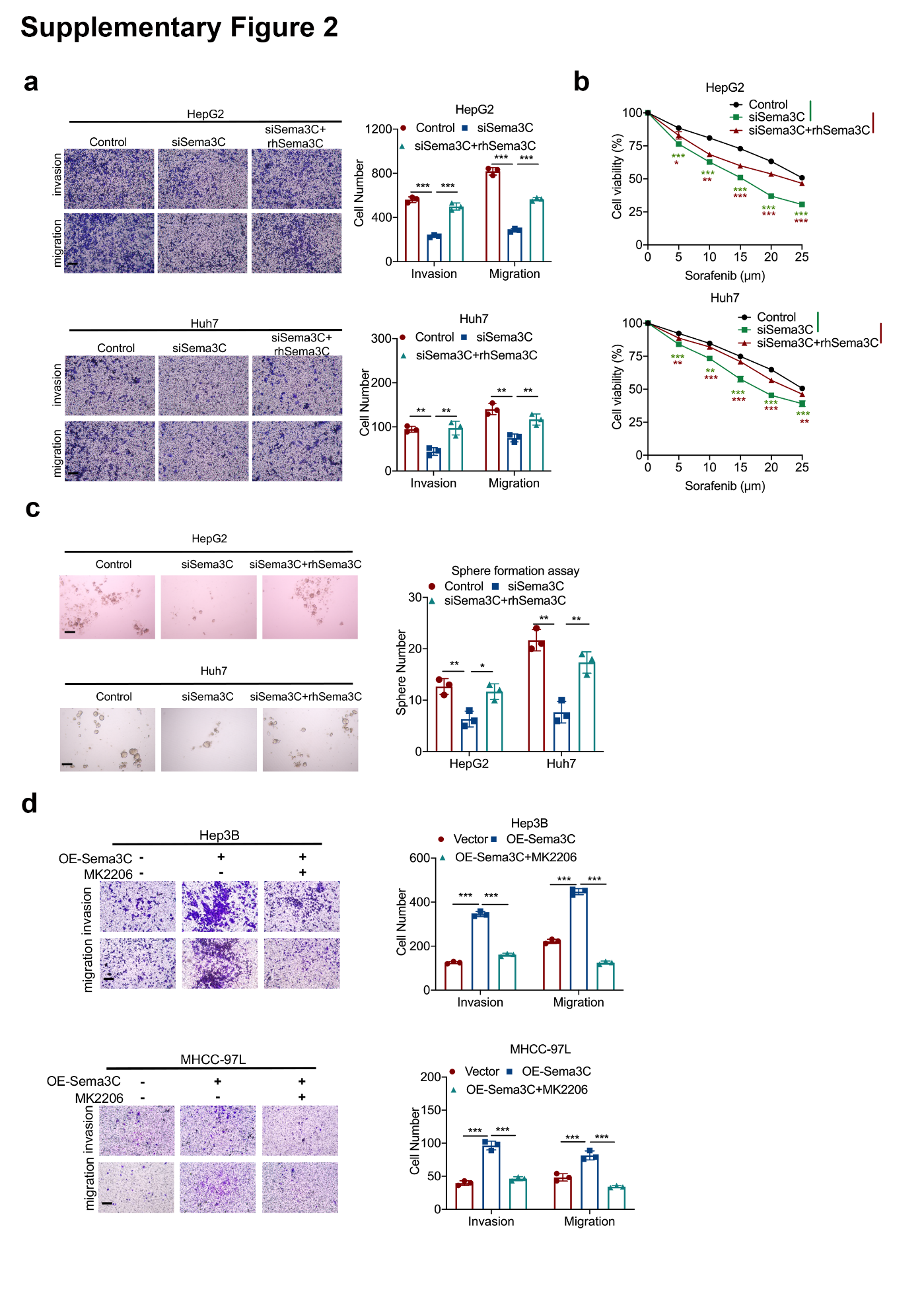


**Supplementary Figure S2. Effects of secretory Sema3C on HCC cells.**

(a-c) Sema3C was knocked down in HCC cells, and 300 ng/ml of rhSema3C was added to stimulate HCC cells for 48 hours. Transwell was used to detect the migration and invasion ability of cells in each group. Scale bar, 200 μm (a). MTT assay was used to determine the drug resistance of the HCC cells (b). The self-renewal ability was detected by sphere formation assay, Scale bar, 100 μm (c).

(d) Sema3C-overexpressing Hep3B and MHCC-97L cells were treated with MK2206 (an AKT inhibitor) as indicated, and cell migration and invasion were evaluated by a transwell assay. Scale bar, 200 μm. rhSema3C, recombinant human Sema3C. Data are presented as means ± SD. *p <0.05, **p <0.01, ***p <0.001.


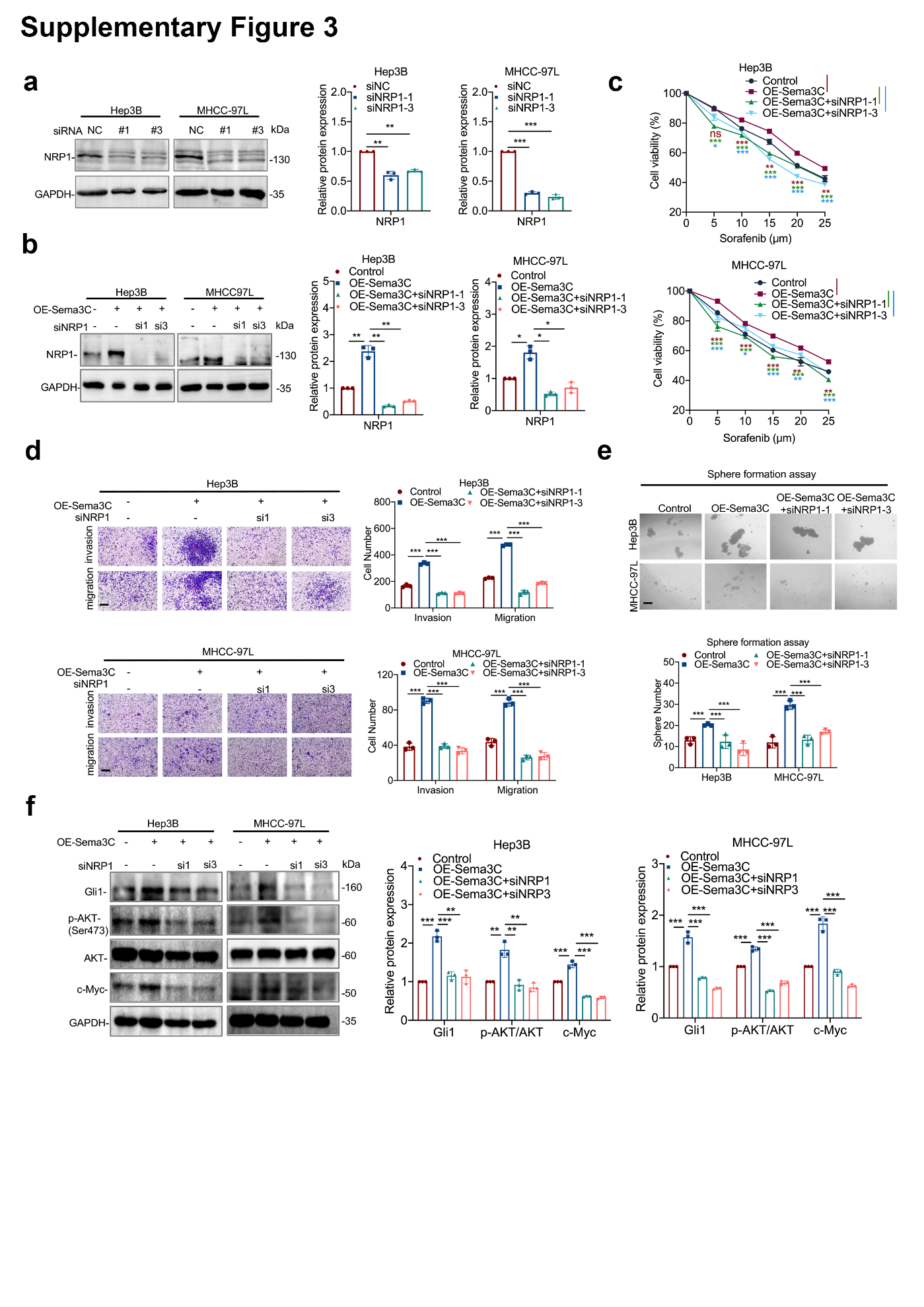


**Supplementary Figure S3. NRP1 mediates the effect of Sema3C in HCC cells.**

(a) Analysis of NRP1 expression by western blotting following siRNA-based NRP1 knockdown in Hep3B and MHCC-97L cells.

(b-f) Hep3B and MHCC-97L cells were transfected with OE-Sema3C or siNRP1 as indicated, the expression of NRP1 was detected by western blotting (b). The chemoresistant effect was investigated by an MTT assay (c). The migration and invasion were evaluated by a transwell assay. Scale bar, 200 μm (d). The ability of self-renewal was determined by a sphere formation assay, Scale bar, 100 μm (e). The protein expression levels of Gli1, p-AKT, AKT, and c-Myc were validated by western blotting (f). Data are presented as means ± SD. ns, not significantly; *p <0.05, **p <0.01, ***p <0.001.


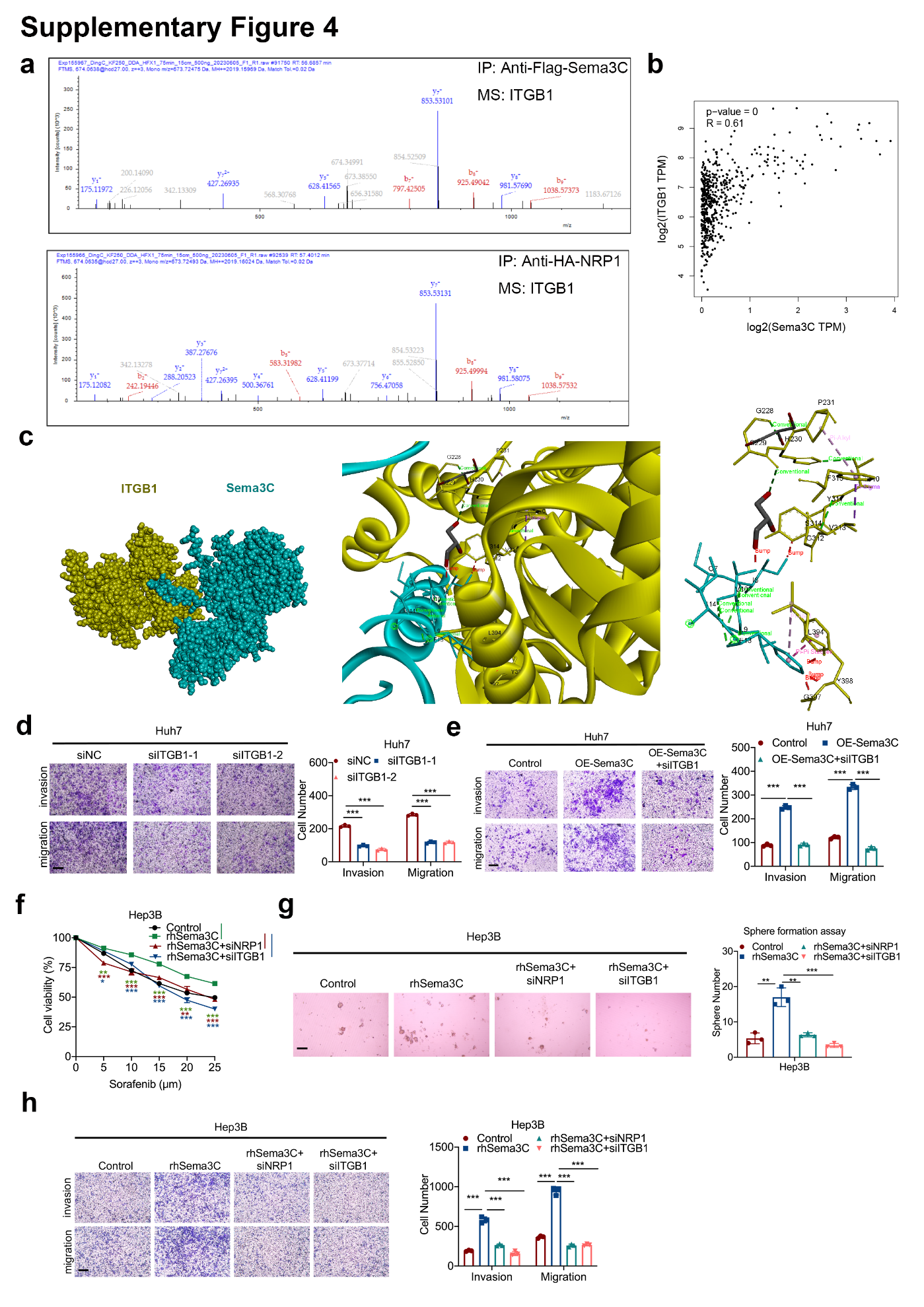


**Supplementary Figure S4. ITGB1 mediates the effect of Sema3C in HCC cells.**

(a) Mass spectrum of ITGB1 detected by mass spectrometry after co-precipitation with flag-Sema3C and HA-NRP1.

(b) Correlation between the levels of the Sema3C and ITGB1 mRNAs in HCC tissues based on TCGA datasets.

(c) The binding model of Sema3C (blue) and ITGB1 (yellow). The residues in Sema3C are depicted as blue sticks while residues in ITGB1 are depicted as yellow sticks.

(d) Huh7 cells were transfected with siITGB1, and the migration and invasion were evaluated by a transwell assay. Scale bar, 200 μm.

(e) Huh7 cells were transfected with OE-Sema3C or siITGB1 as indicated, and the migration and invasion were evaluated by a transwell assay. Scale bar, 200 μm.

(f-h) Hep3B cells were transfected with siNRP, siITGB1 or stimulated with rhSema3C in vitro as indicated. MTT assay was used to detect the chemoresistance (f). Sphere formation assay was conducted to evaluate the self-renewal ability, Scale bar, 100 μm (g). Transwell assay was performed to determine the migration and invasion, Scale bar, 200 μm (h). NC, nontarget control. Data are presented as means ± SD. ns, not significantly; *p <0.05, **p <0.01, ***p <0.001.


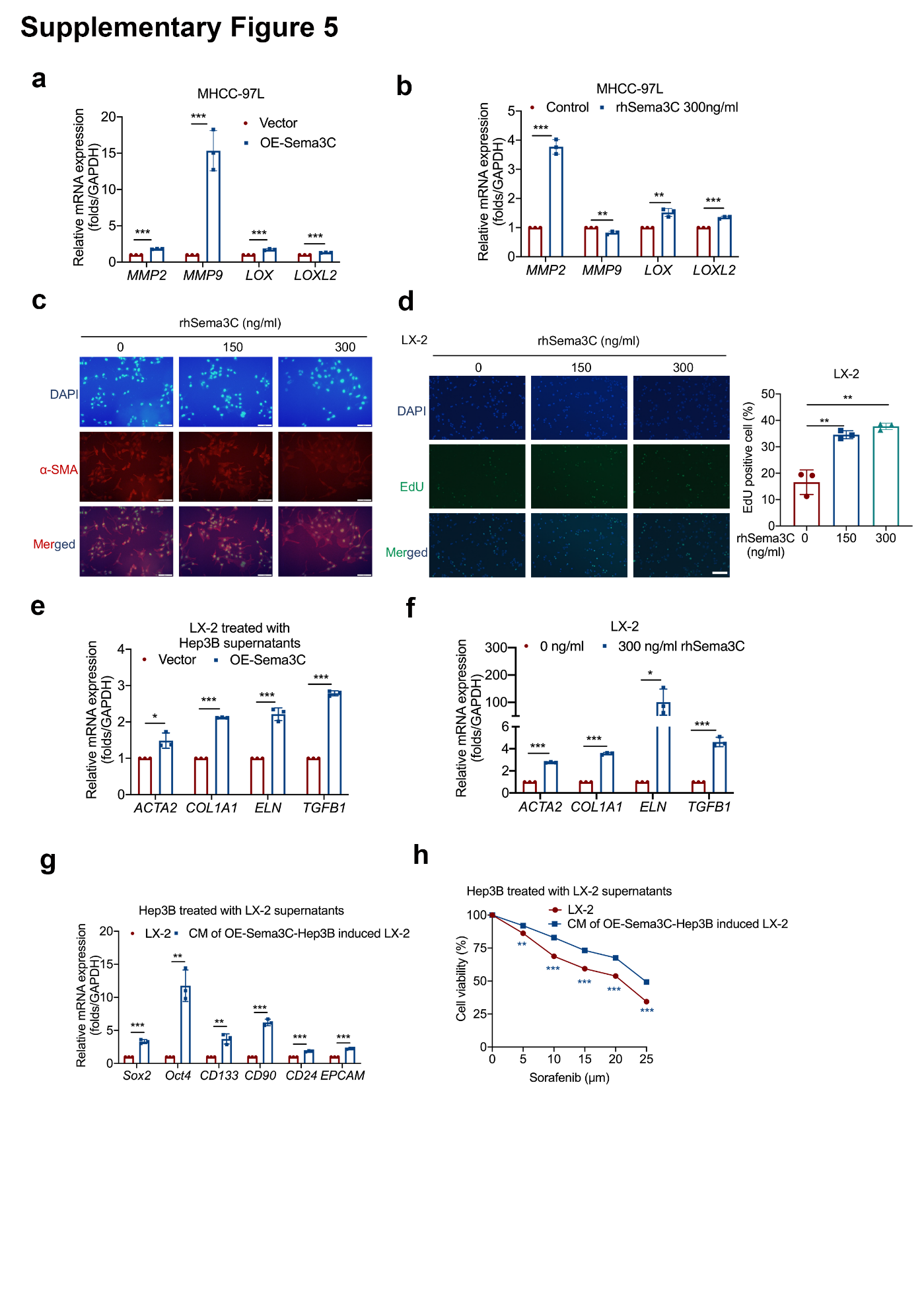


**Supplementary Figure S5. Sema3C promotes the expression of ECM-related genes in HCC cells and collagen-related genes in LX-2 cells.**

(a, b) qRT-PCR showed the expression of MMP2, MMP9, LOX, and LOXL2 genes after overexpression Sema3C or rhSema3C stimulation in MHCC-97L cells in vitro.

(c, d) LX-2 cells were stimulated with a gradient dose of rhSema3C for 48 h in vitro, and morphological changes were detected by immunofluorescence, α-SMA (Red), DAPI (Blue). Scale bar, 200 μm (c). The cell proliferation capacity of LX-2 cells was evaluated using EdU assay. Scale bar, 100 μm (d).

(e, f) qRT-PCR showed that supernatants from Sema3C-overexpressing Hep3B cells (e) or rhSema3C (f) induced increased expression of ACTA2 (encoding α-SMA), collagen-related genes (COL1A1, ELN) and TGFB1 in LX-2 cells.

(g, h) Hep3B cells were treated with different LX-2 cell supernatants, stemness-related genes were detected by qRT-PCR (G), and chemotherapy resistance was detected by MTT assay (H). rhSema3C, recombinant human rhSema3C. Data are presented as means ± SD. ns, not significantly; *p <0.05, **p <0.01, ***p <0.001.


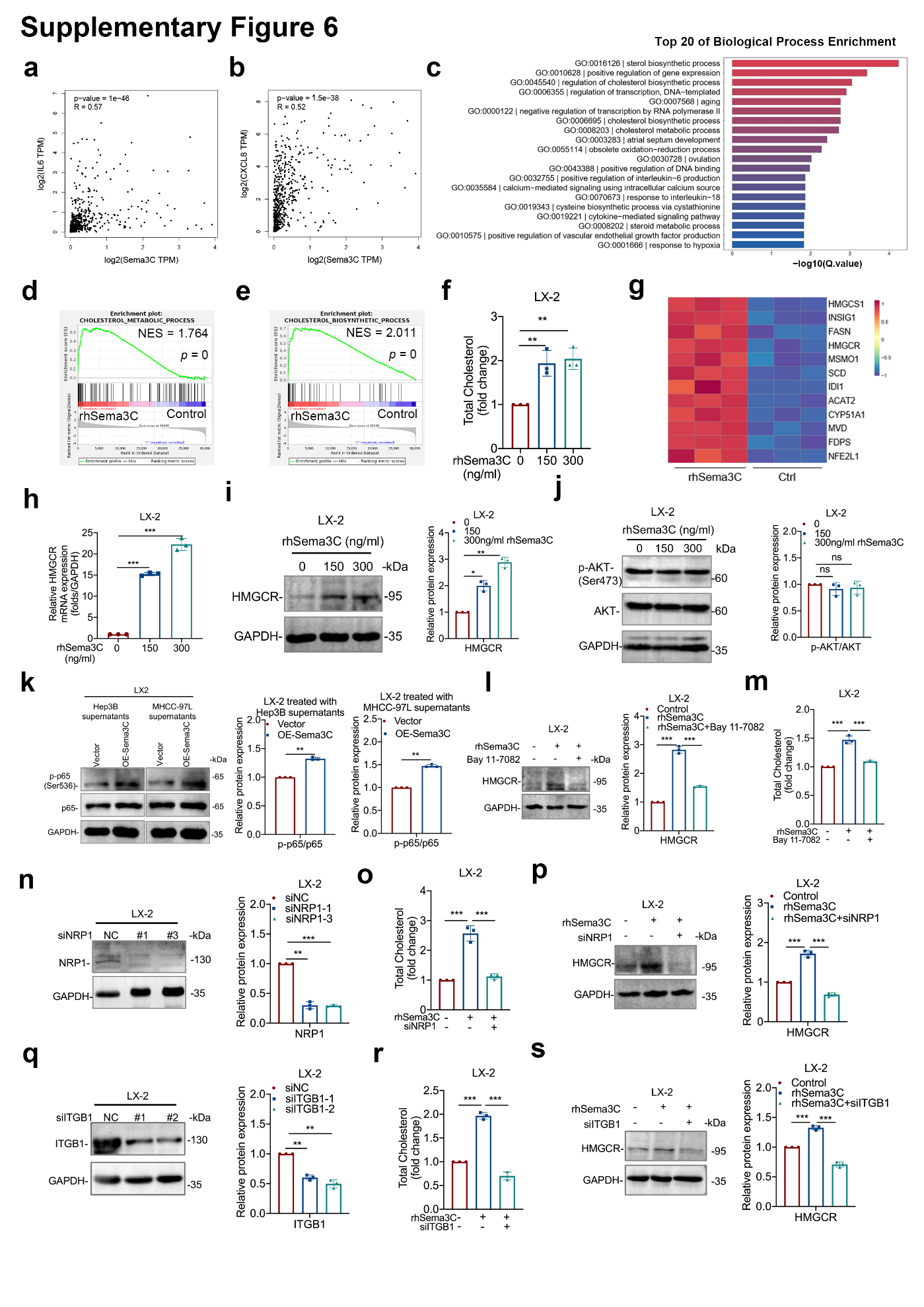


**Supplementary Figure S6. rhSema3C promotes cholesterol synthesis in LX-2 cells by up-regulating HMGCR expression.**

(a, b) Correlation analysis of Sema3C expression and the mRNA levels of IL6 and CXCL8 (IL8) in HCC tissues using TCGA datasets.

(c) Top 20 biological process enrichment of the differentially expressed genes of LX-2 cells treated with rhSema3C or PBS in vitro.

(d, e) GSEA identified an enrichment of genes involved in the cholesterol metabolism process and cholesterol biosynthetic process in rhSema3C-treated LX-2 cells.

(f) LX-2 cells were treated with dosage Sema3C, and the total cholesterol content was detected in vitro.

(g) The heat map showed the differentially expressed genes involved in the regulation of cholesterol metabolism in the rhSema3C treated groups and the control groups.

(h, i) LX-2 cells were treated with dosage Sema3C, and the mRNA and protein level of HMGCR was examined by qRT-PCR (h) and western blotting analysis (i).

(j) Western blotting showed phosphorylated and total AKT protein expression levels in LX-2 cells treated with rhSema3C.

(k) Western blotting showed phosphorylated and total p65 protein expression levels in LX-2 cells treated with supernatants of OE-Sema3C Hep3B or MHCC-97L cells.

(l, m) LX-2 cells were pre-treated with Bay 11-7082 and subsequently stimulated with rhSema3C, HMGCR expression were determined by western blotting (l), and total cholesterol levels were measured (m).

(n) The expression levels of NRP1 were determined by western blotting in NRP1 knockdown LX-2 cells.

(o, p) LX-2 cells were transfected with siINRP1 and subsequently stimulated with rhSema3C, the total cholesterol was measured (o), and the HMGCR expression levels were determined by using western blotting (p).

(q) The expression levels of ITGB1 were determined by western blotting in ITGB1 knockdown LX-2 cells.

(r, s) LX-2 cells were transfected with siIITGB1 and subsequently stimulated with rhSema3C, the total cholesterol was measured (r), and the HMGCR expression levels were determined by using western blotting (s). Data are presented as means ± SD. *p <0.05, **p <0.01, ***p <0.001.


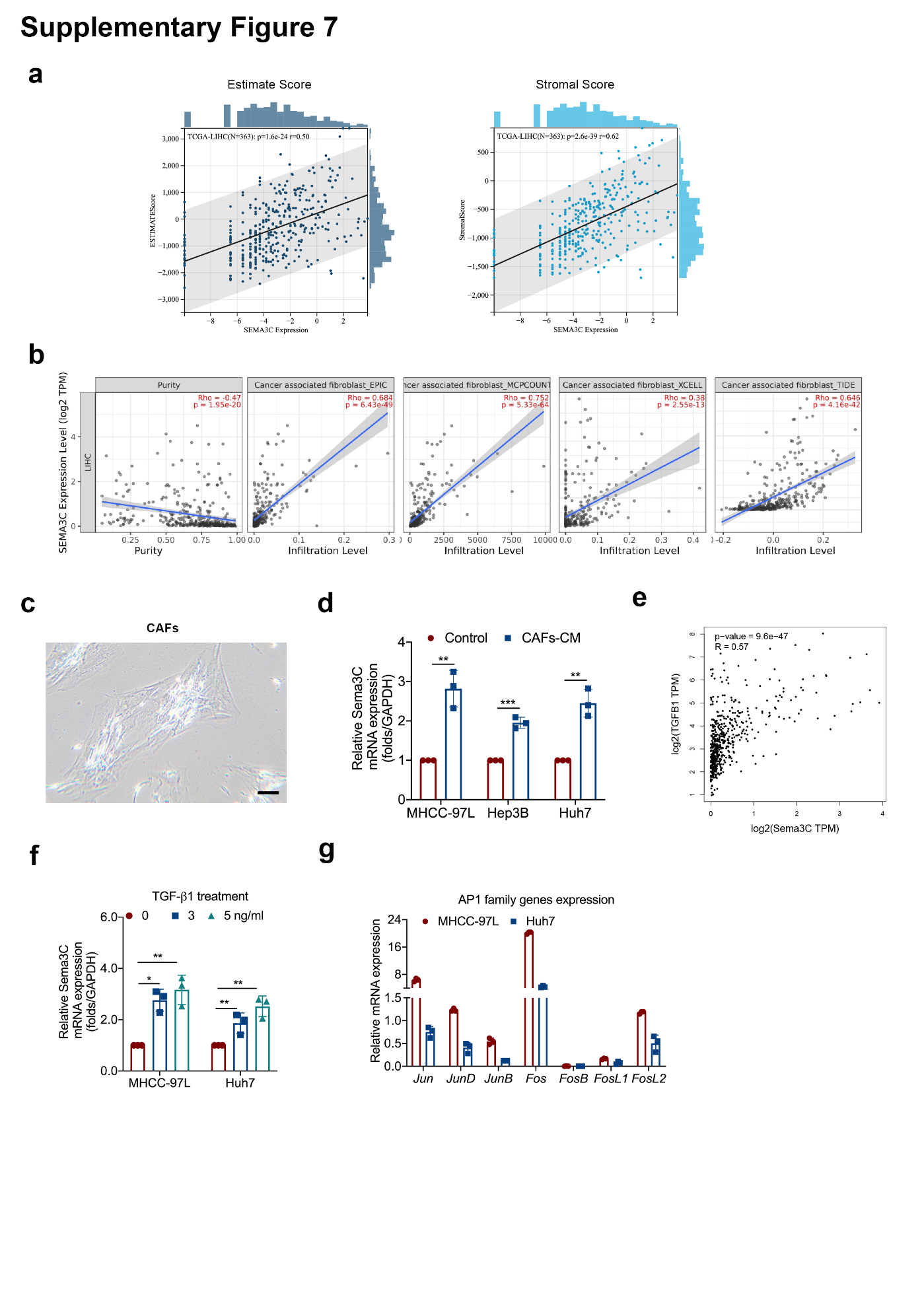


**Supplementary Figure S7. The expression of Sema3C was positively correlated with CAFs infiltration in HCC.**

(a) The correlation between Sema3C and Estimate, Stromal score in HCC tissues was obtained from TCGA datasets.

(b) Correlation between infiltration levels of CAFs and Sema3C expression in HCC tissues was estimated with TIMER2.0 datasets.

(c) Representative brightfield images of CAFs harvested from HCC tumor samples. Scale bar, 10 μm.

(d) qRT-PCR showed that treatment with CAFs-conditioned media (CAFs-CM) significantly increased Sema3C mRNA expression compared with the control in HCC cells.

(e) The correlation analysis showed a positive correlation between Sema3C expression and TGF-β1 expression in HCC from TCGA datasets. CAFs, cancer-associated fibroblasts.

(f) qRT-PCR verified the Sema3C mRNA levels in MHCC-97L and Huh7 cells treated with gradient dose TGF-β1.

(g) The relative mRNA expression of AP-1 family genes in MHCC-97L and Huh7 cells was determined by qRT-PCR. Data are presented as means ± SD. *p <0.05, **p <0.01, ***p <0.001.

| **Supplementary Table S1** |  |  |
| --- | --- | --- |
| **Primers for qRT-PCR** |  |  |
| **Gene name (Human)** | **Forward (5’-3’)** | **Reverse (5’-3’)** |
| *Sema3C* | TTTGCGTGTTGGTTGGAGTAT | TCCTGTAGTCTAAAGGATGGTGG |
| *Sema3C* (for ChIP) | GGTCACCACCCTGTCTTTGG | AGGGCAAATTGTGAGCCTTC |
| *Sox2* | TACAGCATGTCCTACTCGCAG | GAGGAAGAGGTAACCACAGGG |
| *OCT4* | GGGAGATTGATAACTGGTGTGTT | GTGTATATCCCAGGGTGATCCTC |
| *Nanog* | TGAACCTCAGCTACAAACAG | TGGTGGTAGGAAGAGTAAAG |
| *EpCAM* | ATAACCTGCTCTGAGCGAGTG | TGCAGTCCGCAAACTTTTACTA |
| *CD133* | GGCCCAGTACAACACTACCAA | ATTCCGCCTCCTAGCACTGAA |
| *CD90* | TCACCCATCCAGTACGAGTTC | GGAGCGGTATGTGTGCTCAG |
| *CD24* | CTCCTACCCACGCAGATTTATTC | AGAGTGAGACCACGAAGAGAC |
| *Gli1* | GGGTGCCGGAAGTCATACTC | GCTAGGATCTGTATAGCGTTTGG |
| *Gli2* | CCCCTACCGATTGACATGCG | GAAAGCCGGATCAAGGAGATG |
| *c-Myc* | GTCAAGAGGCGAACACACAAC | TTGGACGGACAGGATGTATGC |
| *CCND1* | GCTGCGAAGTGGAAACCATC | CCTCCTTCTGCACACATTTGAA |
| *ACTA2* | CGTGCTGGACTCTGGAGATG | GCCCATCAGGCAACTCGTAA |
| *COL1A1* | GAGGGCCAAGACGAAGACATC | CAGATCACGTCATCGCACAAC |
| *ELN* | GCAGGAGTTAAGCCCAAGG | TGTAGGGCAGTCCATAGCCA |
| *TGFB1* | CAATTCCTGGCGATACCTCAG | GCACAACTCCGGTGACATCAA |
| *MMP2* | TACAGGATCATTGGCTACACACC | GGTCACATCGCTCCAGACT |
| *MMP9* | AGACCTGGGCAGATTCCAAAC | CGGCAAGTCTTCCGAGTAGT |
| *LOX* | TTCTTACCCAGCCGACCAAGATA | GTGTTGGCATCAAGCAGGTCA |
| *LOXL2* | CTCCTCCTACGGCAAGGGA | ATGTCCTCCACCTGGATATTCA |
| *IL6* | ACTCACCTCTTCAGAACGAATTG | CCATCTTTGGAAGGTTCAGGTTG |
| *IL8* | ACTGAGAGTGATTGAGAGTGGAC | AACCCTCTGCACCCAGTTTTC |
| *HMGCR* | TGATTGACCTTTCCAGAGCAAG | CTAAAATTGCCATTCCACGAGC |
| *Jun* | CCTTGAAAGCTCAGAACTCGGAG | TGCTGCGTTAGCATGAGTTGGC |
| *JunB* | CGATCTGCACAAGATGAACCACG | CTGCTGAGGTTGGTGTAAACGG |
| *JunD* | ATCGACATGGACACGCAGGAGC | CTCCGTGTTCTGACTCTTGAGG |
| *Fos* | GCCTCTCTTACTACCACTCACC | AGATGGCAGTGACCGTGGGAAT |
| *FosB* | TCTGTCTTCGGTGGACTCCTTC | GTTGCACAAGCCACTGGAGGTC |
| *FosL1* | GGAGGAAGGAACTGACCGACTT | CTCTAGGCGCTCCTTCTGCTTC |
| *FosL2* | AAGAGGAGGAGAAGCGTCGCAT | GCTCAGCAATCTCCTTCTGCAG |
| *GAPDH* | GGAGCGAGATCCCTCCAAAAT | GGCTGTTGTCATACTTCTCATGG |
| **Gene name (Mouse)** | **Forward (5’-3’)** | **Reverse (5’-3’)** |
| *Sema3C* | GCCAGCATCAACAATCAAAGTT | TCTGAATCACCCGGACGAAAT |
| *GAPDH* | AGGTCGGTGTGAACGGATTTG | TGTAGACCATGTAGTTGAGGTCA |

| **Supplementary Table S2** |  |  |
| --- | --- | --- |
| Antibodies used in this study |  |  |
| Antibodies | Company | Catalog |
| Sema3C | Abmart | TD8610S |
| Sema3C | R&D | MAB1728 |
| Gli1 | Proteintech | 66905-1-Ig |
| Phospho-Akt (Ser473) | Cell Signaling Technology | #4060 |
| Akt (pan) | Cell Signaling Technology | #4691 |
| c-Myc | Proteintech | 67447-1-Ig |
| β-catenin | Proteintech | 51067-2-AP |
| p-YAP | Cell Signaling Technology | #13008 |
| GAPDH | Proteintech | 60004-1-Ig |
| β-actin | Proteintech | 66009-1-Ig |
| c-Jun | Cell Signaling Technology | #9165 |
| Phospho-c-Jun | Cell Signaling Technology | #3270 |
| c-Fos | Cell Signaling Technology | #2250 |
| Phospho-c-Fos (Ser32) | Cell Signaling Technology | #5348 |
| Collagen Type I | Proteintech | 14695-1-AP |
| a-SMA | Servicebio | GB111364 |
| Phospho-NF-κB p65 (Ser536) | Cell Signaling Technology | #3033 |
| NF-κB p65 | Cell Signaling Technology | #4764 |
| HMGCR | Santa Cruz | sc-271595 |
| NRP1 | Proteintech | 60067-1-Ig |
| ITGB1 | Proteintech | 12594-1-AP |
| HRP conjugated goat anti- rabbit IgG | Servicebio | GB23303 |
| HRP conjugated goat anti- mouse IgG | Servicebio | GB23301 |
